# Efficacy and safety of ultrasound-guided nerve blocks in elderly surgical patients: a meta-analysis

**DOI:** 10.3389/fmed.2025.1580172

**Published:** 2025-08-18

**Authors:** Rong Wang, Jinlong Ju, Xiaoyi Pang

**Affiliations:** ^1^Department of Anesthesiology, Mianyang 404 Hospital, Mianyang, Sichuan, China; ^2^Department of Anesthesiology, Sichuan Science City Hospital, Mianyang, Sichuan, China

**Keywords:** elderly, clinical effects, safety, meta-analysis, ultrasound-guided nerve blocks

## Abstract

**Objective:**

This meta-analysis investigates the efficacy and safety of ultrasound-guided nerve block in geriatric surgical patients, a crucial endeavor for optimizing anesthetic protocols and enhancing perioperative medical outcomes and prognostic indices in this vulnerable patient cohort.

**Methods:**

A comprehensive literature search was conducted in major bibliographic databases, including Web of Science, PubMed, The Cochrane Library, Embase, CNKI, Wan Fang, and VIP, to identify studies investigating the utilization of ultrasound-guided nerve blocks in elderly surgical patients. The search encompassed publications from database inception to January 2025. Two independent reviewers meticulously screened the retrieved literature, extracted relevant data, and assessed the risk of bias inherent in the included studies. Subsequently, a meta-analysis was performed to synthesize the findings.

**Results:**

Twenty-five randomized trials were included in the analysis, The meta-analysis showed that, Compared with the control group, Ultrasound-guided nerve block Blocking effective rate ([RR = 1.21 (1.11, 1.31), *P* < 0.001], time of onset of sensory nerve block [SMD = −2.76 (−3.67, −1.85), *P* < 0.001] and Duration [SMD = 2.52 (1.86, 3.18), *P* < 0.01]; Time to onset of motor nerve block [SMD = −1.94 (−2.68,−1.19), *P* < 0.001], and Duration [SMD = 1.11 (0.24, 1.98), *P* = 0.01] has a good effect. After the ultrasound-guided nerve block group, 4 [SMD = −1.68 (−2.68, −0.68), *P* < 0.001], 8 [SMD = −0.99 (−1.66, −0.32), *P* < 0.001], 12 [SMD = −1.03 (−1.0.48, −0.58), *P* < 0.001], 24 [SMD = −1.56 (−2.36, −0.75), *P* < 0.001] hours had all lower VAS scores than the control group. The Adverse reaction rate in the experimental group was 0.35 times that of the control group [RR = 0.35 (0.23, 0.55), *P* < 0.001] and no significant difference between the operating groups [SMD = 0.25 (−0.36, 0.86), *P* = 0.43]. Significant publication bias was found except for the duration of the motor nerve block and surgery time. Sensitivity analysis confirmed the stability of the results.

**Conclusion:**

Ultrasound-guided nerve blocks constitute an efficacious and safe anesthetic modality for elderly surgical patients. These findings have significant implications for the refinement of anesthetic protocols within the geriatric population, with the potential to enhance perioperative medical quality and patient outcomes. The evidence presented in this meta-analysis provides a robust foundation for the widespread adoption of ultrasound-guided nerve blocks in clinical practice for elderly surgical patients.

## 1 Introduction

The burgeoning elderly population has resulted in a significant increase in the number of geriatric surgical patients (1). Owing to their unique physiological characteristics, including organ system decline and the concomitant presence of multiple chronic diseases, elderly patients present heightened demands for anesthetic tolerance and safety (2, 3). The judicious selection of anesthetic techniques is paramount for optimizing perioperative outcomes, facilitating postoperative rehabilitation, and minimizing the incidence of complications in elderly surgical patients (4).

Nerve block, a widely employed anesthetic modality, constitutes a cornerstone of surgical anesthesia and postoperative pain management (5). Notably, elderly patients derive the greatest benefit from successful regional nerve blocks, as this approach markedly reduces the risk of postoperative delirium—a common and severe complication in this population associated with prolonged hospital stays, increased mortality, and long-term cognitive decline. Conventional nerve block techniques predominantly depend upon the identification of anatomical surface landmarks or the utilization of nerve stimulators (6). Nevertheless, these methodologies exhibit inherent limitations, including imprecise positioning and an inconsistent success rate (7). Notably, these challenges may be exacerbated in geriatric patients due to age-related alterations in anatomical structures and a concomitant decline in physiological function.

The advent of ultrasound-guided nerve block technology has ushered in a new era of innovation within the field of nerve block. This technique constitutes an anesthetic modality that enables the precise localization of analgesia through the real-time or visually guided administration of the anesthetic agent (8). This approach enables direct visualization of both the nerve and the puncture needle, thereby markedly enhancing puncture accuracy and mitigating the risks inherent in blind puncture procedures (9). For elderly patients, this precision is crucial not only for block success but also for avoiding complications that could precipitate delirium, such as excessive sedation or respiratory depression from rescue analgesics. This visualization facilitates the identification of perivascular anatomy, thereby preventing inadvertent needle penetration of blood vessels. This mitigates the risk of local anesthetic systemic toxicity and minimizes the likelihood of iatrogenic injury to vital structures, including nerves and blood vessels, ultimately enhancing patient safety (10). The ultrasound-guided nerve block technique has an extremely wide range of applications. It exhibits utility in both anesthetic and postoperative analgesic contexts for a diverse range of surgical procedures, encompassing upper extremity, lower extremity, and abdominal surgeries. Furthermore, it demonstrates efficacy in the management of various neuropathic pain conditions (11–13). Owing to its multifaceted benefits, this technique has found widespread application across diverse medical domains, with particularly promising outcomes observed in geriatric surgical patients (14, 15).

Nevertheless, despite the theoretical promise of ultrasound-guided nerve block technology in geriatric surgical patients, assessing its clinical efficacy presents a multifaceted and heterogeneous challenge. Some studies have demonstrated that ultrasound-guided nerve blocks can significantly reduce postoperative pain intensity in surgical patients, decrease opioid consumption, and lower the incidence of complications—including delirium, which is directly mitigated by reduced opioid use—thereby improving patient prognosis (1, 16); some studies point out that this technology may have some technical difficulties in practice, and the analgesic effect for some specific types of surgery or patient groups, the effect may not be ideal, and may even increase the risk of some complications (17, 18).

Therefore, a rigorous and comprehensive systematic review of relevant literature is imperative to accurately assess the efficacy and safety profile of ultrasound-guided nerve blocks in geriatric surgical patients. This meta-analysis endeavors to systematically synthesize and critically appraise the existing evidence pertaining to the safe and effective implementation of ultrasound-guided nerve blocks within the context of geriatric surgical procedures, thereby providing a more robust foundation for clinical decision-making.

## 2 Methods

### 2.1 Search strategies

This study was a systematic review and meta-analysis using the Preferred Reporting Project (PRISMA) report list (19). The meta-analysis was carefully constructed according to the PICO (Patient, Intervention, Comparison, Outcome) framework, describing the key aspects as follows: Patient (P) was a geriatric surgical patient. The intervention studied (I) was the ultrasound-guided nerve block. Comparison (C) relates to other forms of anesthesia other than ultrasound-guided nerve block. For outcome (O), our primary focus was on the effect on postoperative wound pain, as well as secondary outcomes such as the success rate of nerve blocks, time to onset, duration of analgesia, and frequency of complications.

We searched the relevant literature on 2 January 2025 in various electronic databases: PubMed, Web of Science, Cochrane Library, CNKI, Wang Fang, VIP, and Embase, without imposing any time limits. There are no restrictions on language. In addition, a manual review of the reference list of relevant articles was conducted to identify any further potential records. The search strategy incorporates key terms including: “Ultrasound-guided nerve block,” “US-guided SNB,” “nerve block,” “Ultrasound-guided scalp nerve block,” “Block,” “Nerve Blocks,” “Blockade,” “Elderly patients,” “old people,” “geriatrics,” “geriatrist,” “Geriatric Anesthesia.” These terms have been deliberately chosen to capture the broad scope of the PICO framework and to ensure a comprehensive compilation of relevant studies for this meta-analysis, for which specific search strategies are detailed in Supplementary Table 1 in Supplementary File 1.

### 2.2 Inclusion criteria and exclusion criteria

#### 2.2.1 Inclusion criteria

   1.  Study Design: Randomized controlled trials (RCTs) and observational studies assessing the efficacy of ultrasound-guided nerve block.
   2.  Population: Elderly patients (age ≥=65 years or older).
   3.  Interventions: Studies must involve the use of ultrasound-guided nerve block as an intervention.
   4.  Comparison: Other forms of anesthesia other than ultrasound-guided nerve block.
   5.  Outcomes: The primary outcome must include the assessment of postoperative wound pain. Secondary outcomes should include the success rate of nerve block, onset time, duration of analgesia, and incidence of complications.

#### 2.2.2 Exclusion criteria

   1.  Non-Clinical Studies: Laboratory-based or animal studies, case reports, reviews, editorials, and letters to the editor.
   2.  Non-Adult Population: Studies involving pediatric patients or patients under 65.
   3.  Non-Ultrasound-Guided Techniques: Studies that do not specifically use ultrasound guidance for the nerve block.
   4.  Incomplete Data: Studies with incomplete outcome data relevant to the primary and secondary outcomes as defined.
   5.  Duplicate Publications: Studies that are duplicate publications or sub-studies of included trials.

### 2.3 Data extraction

In our meta-analysis, literature screening and data extraction were rigorously performed by two independent evaluators, followed by a thorough cross-verification to ensure accuracy and precision. Discrepancies encountered during this process were resolved through collaborative discussions between the evaluators, with the involvement of a third-party reviewer when consensus could not be reached. The extracted data encompassed several key elements: the author(s) of each study, the publication year of the study, and specific characteristics of the included studies such as sample size, anesthesia methods, the site of nerve block, and the types and dosages of drugs used for the nerve block. The primary outcome measure extracted was the success rate of the nerve block within 30 min. Secondary outcome measures included the onset time of the block, the duration of the block’s effect, the duration of the block procedure, and the incidence rate of complications.

### 2.4 Quality of individual studies

The Cochrane Manual of Systematic Evaluators 5.1.0 (20) was used as the criterion for literature quality evaluation, which included random allocation methods, hiding of allocation schemes, implementation of blind methods, integrity of outcome data, selective reporting of findings, and other sources of bias. The judgments included “low risk of bias,” “unknown risk of bias,” and “high risk of bias.”

### 2.5 Statistical analyses

Statistical analysis was performed using Stata 16 software. The χ^2^ test was used to analyze the heterogeneity among the studies. If *P* > 0.1 and I^2^ < 50%, there was no statistical heterogeneity among the studies. A fixed-effects model was initially employed for the meta-analysis. However, evidence of significant heterogeneity among the included studies was observed. Consequently, a random-effects model was adopted to account for this between-study variability. Subgroup analyses were subsequently conducted to explore potential sources of heterogeneity. In the absence of identifiable sources of heterogeneity, descriptive analyses were performed to characterize the observed variability. Sensitivity analyses were then implemented to assess the robustness of the findings. Relative risk (RR) and 95% confidence intervals (CI) were used to estimate clinical efficacy, while continuous data were presented as Standardized Mean Difference (SMD) and 95% confidence intervals. An inverted funnel diagram and Begg’s test were used for publication bias analysis.

## 3 Results

### 3.1 Selected studies

**FIGURE 1:**
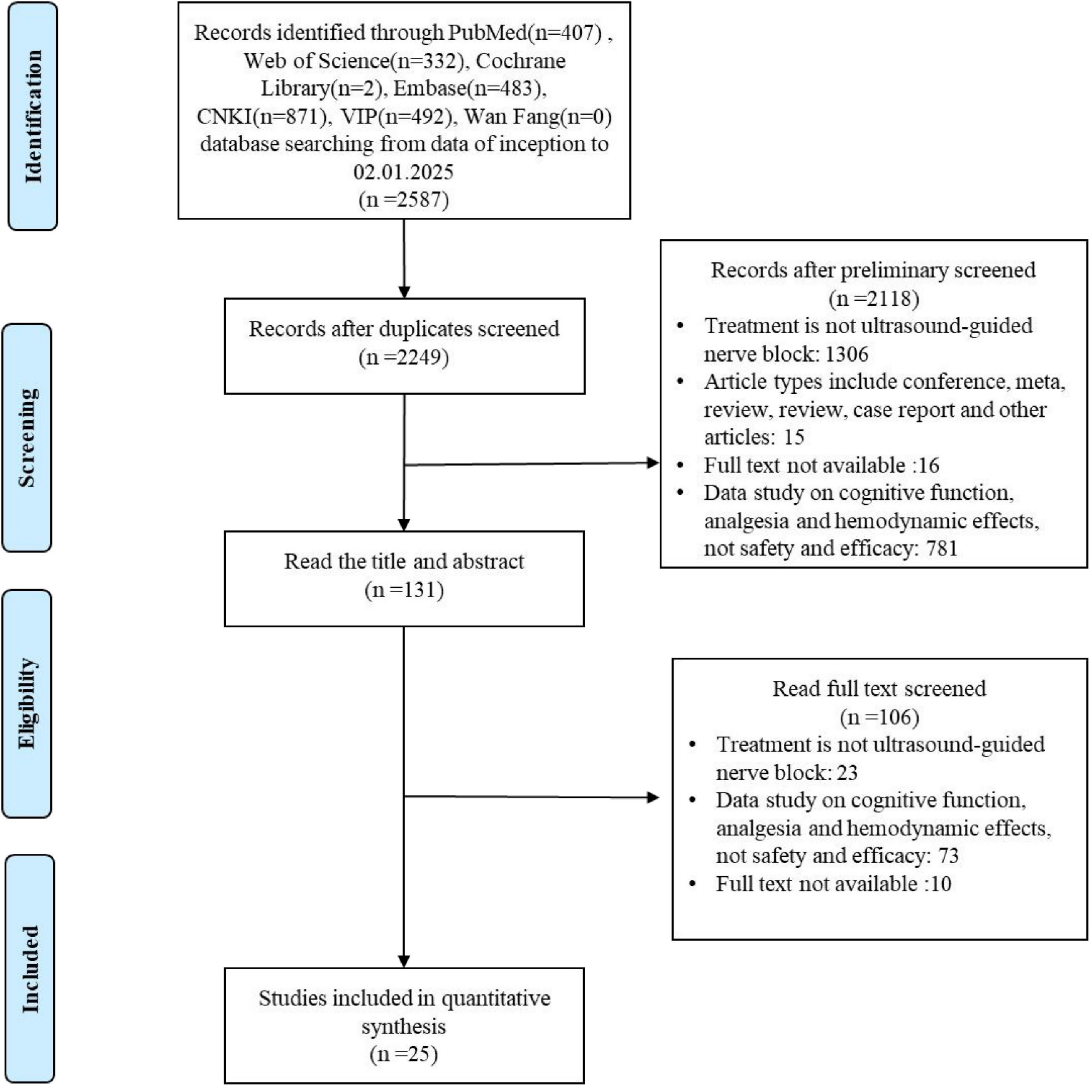
The literature screening process of the meta-analysis.

A stepwise approach was employed to select eligible studies. All identified articles were exported to EndNote X9 Citation Manager. An initial screening of titles and abstracts resulted in the exclusion of 2,587 records. Furthermore, 338 duplicate entries were removed. Following a comprehensive evaluation of the remaining articles, 2,118 records were excluded. Ultimately, 25 articles met the inclusion criteria after a full-text review. The study selection process is depicted in Figure 1.

### 3.2 Study characteristics

The basic characteristics of the included works of literature are shown in Table 1. All the 25 included studies were retrospective studies or prospective studies. The inclusion and exclusion criteria and the evaluation criteria for each indicator are detailed in Supplementary Table 2 in Supplementary File 1.

**TABLE 1:** Characteristics of included studies.

First author	Year	Country	Type of operation	The anesthetic		Guidance mode				Sample (Exp/Con.)	Gender (males/Females)		Age (year)		Complication incidence
				Drugs and concentrations	Dosage	Exp.	Specific locations	Con.	Specific locations		Exp.	Con.	Exp.	Con.	
Zhang Weiqi (21)	2019	China	Lower extremity orthopedic surgery	1% lidocaine	Not clear	Ultrasound-guided lower back plexus sciatic nerve block anesthesia	From the midline of the spine to the transverse process	Epidural anesthesia	From the center of the spine to the transverse process	16/16	9/7	8/8	69.17 ± 3.01	68.33 ± 2.43	Not clear
Bian Yupu (22)	2020	China	Artificial knee replacement	0.5% ropivacaine	50 mL	Ultrasound-guided lower back plexus sciatic nerve block anesthesia	Positioning of the lumbar plexus and sciatic nerve	Epidural anesthesia	Epidural space	49/49	26/23	25/24	68.24 ± 6.68	68.31 ± 5.31	Dyspnea, Nerve injury, Urinary retention
Cao Yongchao (23)	2021	China	Upper extremity operation	0.375% ropivacaine	20 mL	Ultrasound-guided nerve block anesthesia was performed	Between the scalenus medius muscle and the superior/middle/ inferior trunks of the brachial plexus	Brachial plexus block anesthesia	Above the thyrohyoid bone, within the interscalene groove between the anterior and middle scalene muscles	42/38	22/20	20/18	67.58 ± 5.23	67.34 ± 5.11	Hematoma, Pneumothorax, Nausea, Vomiting
Wang Yuansheng (24)	2016	China	Upper extremity operation	0.5% ropivacaine	30 mL	Ultrasound-guided descending intermuscular sulcus brachial plexus block	Between the anterior and middle scalene muscles	Neurostimulator localization of descending intermuscular groove brachial plexus block	The intersection line between the interscalene groove and the level of the cricoid cartilage	20/20	11/9	10/10	68 ± 7	66 ± 11	Hematoma, Block failure
Wang Zepeng (25)	2022	China	Senile patellar fracture	0.5% bupivacaine	Not clear	Ultrasound-guided femoral nerve block	Inguinal region	Combined epidural anesthesia	A puncture was performed at the patient’s L3-L4 intervertebral space	38/38	20/18	19/19	67.8 ± 1.5	67.7 ± 1.3	Urinary retention, Headache, Nausea and vomiting
Deng Guohua (26)	2022	China	Elderly knee replacement	Dezocine, Sufentanil	Not clear	Ultrasound guided femoral nerve block	Placed 2 cm below the patient’s inguinal ligament	Venous control	Connect the patient-controlled analgesia pump to the peripheral venous access.	40/40	23/17	27/13	65.6 ± 4.63	65.97 ± 4.32	Delirium, Urinary Retention, Nausea and Vomiting, Cognitive Dysfunction
Li Fangqing (27)	2017	China	Upper extremity operation	0.375%L-bupivacaine + 1% lidocaine	20 mL	Ultrasound-guided brachial plexus block anesthesia	At the interscalene brachial plexus	Intermuscular groove brachial plexus block	Puncture is performed within the interscalene groove between the anterior and middle scalene muscles, and above the omohyoid muscle	21/21	12/9	11/10	63.73 ± 1.78	64.17 ± 1.51	Accidental vascular puncture, Nerve injury
Yu Changwei (28)	2018	China	Upper extremity operation	0.375% ropivacaine	20 mL	Ultrasound-guided brachial plexus block by intermuscular groove combined with supraclavicular approach	The superior and middle trunks of the brachial plexus between the anterior and middle scalene muscles, with localization of the brachial plexus posterior-superior to the subclavian artery	Intermuscular groove brachial plexus block	Insert the needle between the anterior scalene muscle and the middle scalene muscle, and perform a blind insertion at 1 cm above the midpoint of the clavicle.	30/30	19/11	17/13	71.32 ± 3.78	72.51 ± 2.53	Vascular puncture, Pneumothorax, Horner’s syndrome
Zhang Xi (29)	2020	China	Unilateral lower extremity orthopedic surgery	Sufentanil 10 μg and midazolium 1 mg	Not clear	Ultrasound-guided nerve block anesthesia	At the iliac crest level, demonstrating the quadratus lumborum, psoas major, erector spinae muscles, and L4 transverse process. Insert the needle 4–5 cm lateral to the L4 spinous process, advancing until the tip reaches the nerve root for perineural drug injection. The sciatic nerve positioned laterally between the biceps femoris and semitendinosus muscles.	Combined epidural anesthesia	The puncture was performed at the patient’s L2-L3 intervertebral space. After entering the dural cavity, a lumbar puncture needle was inserted. Once cerebrospinal fluid was observed flowing out, 12–15 mg of 5% ropivacaine was slowly injected. Subsequently, an epidural catheter was placed, and an appropriate amount of anesthetic was injected as required.	40/40	23/17	24/16	69.73 ± 3.25	68.85 ± 3.4	Nausea, Vomiting, Urinary retention
Deng Bin (30)	2020	China	Single upper extremity orthopedic surgery	0.375% ropivacaine	Not clear	Ultrasound-guided nerve block anesthesia was performed	Trunks of the brachial plexus (formed by C5–T1 nerve roots	Intermuscular groove brachial plexus block	Above the thyrohyoid muscle, within the interscalene groove between the anterior and middle scalene muscles	35/35	19/16	20/15	68.91 ± 4.19	68.96 ± 4.15	Nausea and vomiting, urinary retention, chills
Kwon Young Sil (31)	2020	China	Upper extremity orthopedic surgery	0.375% ropivacaine	30 mL	Ultrasound-guided nerve block anesthesia	Trunks of the brachial plexus (formed by C5–T1 nerve roots	Intermuscular groove brachial plexus block	Above the thyrohyoid muscle, within the interscalene groove between the anterior and middle scalene muscles	33/33	23/10	21/12	69.69 ± 5.67	69.79 ± 5.71	Chills, nausea and vomiting, urinary retention
Zhang Aiping (32)	2020	China	Elderly intertrochanteric fracture of femur	2% lidocaine and 0.5% ropivacaine	Not clear	Ultrasound-guided lumbar plexus combined with sciatic nerve block anesthesia	The puncture point is located approximately 1.5 cm below the midpoint of the line connecting the highest points of the bilateral anterior superior iliac spines (ASIS) and about 4 cm lateral to the midline on the block side.	Subarachnoid block anesthesia combined with epidural anesthesia	Puncture was performed at the L3-L4 lumbar intervertebral space, a spinal needle was inserted, and after cerebrospinal fluid was obtained, the procedure was continued in the subarachnoid space	55/55	27/28	26/29	61–79	61–80	Nausea and vomiting, bradycardia, urinary retention, chills
Yang Xianzhou (33)	2017	China	Lower extremity orthopedic surgery	0.5% ropivacaine	45 mL	Ultrasound-guided lower back plexus sciatic nerve block anesthesia	At L3 level, perform a parasagittal scan lateral to the midline to visualize the interfacial plane between the quadratus lumborum and psoas major muscles. Insert the needle in-plane 4 cm	Lumbar plexus combined with sciatic nerve block anesthesia	Lumbar plexus block: Insert the needle vertically 5 cm lateral to the L4 spinous process, and locate the psoas major muscle space using the loss-of-resistance technique.	50/50	27/23	28/22	73.5 ± 5.8	74.5 ± 6	Not clear
							-lateral to the spinous process, advancing into the psoas compartment. Transverse scan along the line connecting the ischial tuberosity and greater trochanter to identify the sciatic nerve (oval hyperechoic structure). Perform in-plane needle insertion for perineural injection.		-Sciatic nerve block: Insert the needle vertically at the midpoint of the line connecting the ischial tuberosity and the greater trochanter of the femur, and locate using the paresthesia method.						
Chen Rong (34)	2018	China	Lower extremity orthopedic surgery	0.5% ropivacaine	45 mL	Ultrasound-guided lumbar plexus combined with sciatic nerve block anesthesia	Lumbar Plexus Block Localization: At the L4 level, perform a transverse scan to visualize the L4 transverse process, erector spinae, quadratus lumborum, and psoas major muscles. Under ultrasound guidance,	Lumbar plexus combined with sciatic nerve block anesthesia	Lumbar plexus block: Insert the needle vertically 4–5 cm lateral to the L4 spinous process, and locate the psoas major muscle space using the loss-of-resistance technique.	23/23	12/11	13/10	67.2 ± 2.6	66.5 ± 2.4	Lower limb paresthesia, urinary retention, bradycardia
							-perform a paramedian epidural puncture and advance the needle tip adjacent to the target nerve. Sciatic Nerve Block Localization: At the midpoint of the line connecting the ischial tuberosity and greater trochanter, perform a transverse scan to identify the sciatic nerve, then advance the needle adjacent to the nerve for injection.		-Sciatic nerve block: Insert the needle vertically between the ischial tuberosity and the greater trochanter of the femur, and locate using the paresthesia method.						
Yu Chunlei (35)	2019	China	Lower extremity orthopedic surgery	0.5% ropivacaine	Not clear	Ultrasound guided lower back plexus sciatic nerve block anesthesia	Lumbar Plexus Block: Approach: Interfacial plane approach between major lumbar muscles Scanning landmark: Intersection of the spinal midline and the intercristal line	Epidural anesthesia	Puncture point: 5 cm lateral to the midline of the spine at the L4 interspace (determined based on the iliac crest). Needle insertion point: 2 cm below	100/100	57/43	56/44	68.57 ± 2.42	68.64 ± 2.48	Hypotension, bradycardia
							-(connecting the bilateral iliac crest peaks). Needle insertion is performed at any visualized hypoechoic neurovascular space. Sciatic Nerve Block: Approach: Subgluteal approach Scanning zone: Along the ischial tuberosity-greater trochanter line Sonographic identification: Triangular hyperechoic structure (apex superior) representing the sciatic nerve		-the line connecting the greater trochanter of the femur and the sacral horn (for sciatic nerve block).						
Huang Minzhen (36)	2018	China	Lower extremity orthopedic surgery	1% lidocaine + 0.5% ropivacaine	45 mL	Ultrasound-guided lower back plexus sciatic nerve block anesthesia	Lumbar Plexus Block: Ultrasound scan at L4 transverse process → Insert needle paramedially to L4 nerve root Sciatic Nerve Block: Ultrasound	Lumbar plexus sciatic nerve block was used for anesthesia	5 cm lateral to the L4 spinous process, puncture vertically to the psoas major muscle space 2 cm below the line connecting the greater	45/45	25/20	26/19	70.19 ± 1.72	69.31 ± 1.82	Inadequate anesthesia, puncture-related nerve injury, mild pain
							-locate midpoint between ischial tuberosity and greater trochanter → Advance needle to nerve		-trochanter of the femur and the sacral horn, puncture vertically to the paresthesia point						
Yang Jie (37)	2019	China	Lower extremity orthopedic surgery	0.4% ropivacaine	45 mL	Ultrasound-guided lower back sacral plexus block combined with shallow general anesthesia	Lumbar Plexus Block: Ultrasound scan at L4 transverse process → Insert needle paramedially to L4 nerve root Sciatic Nerve Block: Ultrasound locate midpoint between ischial tuberosity and greater trochanter → Advance needle to nerve	Lumbar plexus sciatic nerve combined block anesthesia	1 cm lateral to the L4 spinous process, puncture vertically to the L4 transverse process → then insert the needle 1 cm above the transverse process. Puncture vertically to the paresthesia point at the junction of 2 cm below the femur and the sacrum.	31/31	16/15	17/14	68.3 ± 2.1	68.7 ± 2.4	Mild pain, inadequate anesthesia
Dong Dalong (38)	2019	China	Total hip replacement	0.5% ropivacaine	Not clear	Ultrasound-guided lower back sacral plexus block combined with shallow general anesthesia	4–5 cm lateral to the L3–4 interspace → ultrasound shows an “oval hyperechoic mass within the psoas major fascia” The medial 1/3 of the line	Combined epidural anesthesia	Puncture at the L2–3 or L3–4 intervertebral space (routine spinal anesthesia) without independent sacral plexus block (relying	40/40	23/17	22/18	75.6 ± 6.8	74.3 ± 5.2	Nausea and vomiting, headache, agitation, hypotension
							-connecting the greater trochanter of the femur and the posterior superior iliac spine → nerve bundle between the sacrum and ilium		-on spinal anesthesia diffusion)						
Cheng Yan (39)	2017	China	Unilateral hip replacement	0.5% ropivacaine	Not clear	Ultrasound guided lower back sacral plexus combined nerve block group	Puncture lateral to the L2–3 interspace → ultrasound shows “battlement-like” transverse processes → the needle tip is advanced 1.5–2.0 cm deep The medial 1/2 of the line connecting the greater trochanter of the femur and the posterior superior iliac spine → the sacral plexus is located by translating the probe posteriorly along the ilium Convex ultrasound probe (2–5 MHz) with real-time guidance	Combined epidural anesthesia	Puncture at the L1–2 intervertebral space → place the epidural catheter 3 cm into the epidural space. No independent sacral plexus block (relying on the spread of the anesthetic plane).	40/40	NA	NA	NA	NA	Hypotension, urinary retention, nausea and vomiting, nerve injury, local anesthetic systemic toxicity (LAST)
Wei Nan Fu (40)	2017	China	Fracture of femoral neck	0.5% ropivacaine	Not clear	Ultrasound guided femoral nerve combined with sciatic nerve block	Lateral to the pulsation point of the femoral artery at the midpoint of the inguinal region → ultrasound short-axis scanning of the nerve bundle Lateral to the line connecting the posterior superior iliac spine and the ischial tuberosity → transverse positioning of the nerve with the probe	Combined epidural anesthesia	Puncture at the L2–3 interspace using anatomical landmarks	76/76	41/35	40/36	75.58 ± 3.18	75.23 ± 3.21	Nausea and vomiting, low back pain, headache
Kateryna Bielka (41)	2021	Ukraine	Fracture of proximal femur	Bupivacaine	Not clear	Ultrasound-guided PCB	The needle tip is placed within the psoas major fascia, closely adjacent to the L2-L3 transverse process interspace.	Subarachnoid block	Puncture at the L3–4 intervertebral space	30/30	21/9	21/9	72(68–73)	72 [70–73]	Hypotension, myocardial injury after non-cardiac surgery (MINS), nausea and vomiting, delirium
Yan Tang (42)	2023	China	Hip fracture surgery	0.375% ropivacaine	Not clear	Ultrasound-guided PENG block	The probe is placed transversely along the line connecting the anterior inferior iliac spine and the pubic ramus.	Intravenous	\	20/21	4/16	3/18	76.45 ± 4.56	78.67 ± 5.92	Nausea and vomiting
Liang Jin (43)	2020	China	Esophagectomy	0.375% ropivacaine	Not clear	Ultrason-guided PVB	The junction of the transverse process and the rib	PCA	\	84/83	40/44	37/46	70.8 ± 5.2	71.4 ± 5.6	Postoperative atelectasis, nausea and vomiting, pruritus, delirium
Qiu Dongjie (44)	2023	China	The surgery of thoracoscopic lobectomy	0.375% ropivacaine	Not clear	Ultrason-guided TPVB	Surgical side T5 intervertebral space (single injection covers T4-T6 dermatomes)	TPVB	\	103/105	59/44	67/38	68 ± 5	69 ± 4	Hypotension, respiratory depression, atelectasis, nausea and vomiting, delirium
Jianhong Hao (45)	2019	China	Scheduled for hipfracture surgery	Ropivacaine	Not clear	Ultrasound-guided lower back plexus sciatic nerve block anesthesia	Surgical side T5 intervertebral space (single injection covers T4-T6 dermatomes)	Generic continuous FICB	\	43/42	23/20	23/19	72.3 ± 3.78	72.52 ± 4.26	Delirium

NA means not clear.

### 3.3 Quality assessment

The quality assessment of this study is shown in Table 2.

**TABLE 2:** Quality assessment (Cochrane analysis bias assessment).

Author	Year	The generation of random sequences	Allocation scheme hiding	Blind subjects and staff	Results were evaluated blind	Results The data were incomplete	Selectively report research findings	Other sources of bias
Zhang Weiqi	2019	Bias ambiguity	Bias ambiguity	Bias ambiguity	Bias ambiguity	Low bias	Low bias	Bias ambiguity
Bian Yupu	2020	Low bias	Low bias	Bias ambiguity	Bias ambiguity	Low bias	Low bias	Low bias
Cao Yongchao	2021	Bias ambiguity	Bias ambiguity	Bias ambiguity	Bias ambiguity	Low bias	Low bias	Bias ambiguity
Wang Yuansheng	2016	Low bias	Low bias	Bias ambiguity	Bias ambiguity	Low bias	Low bias	Low bias
Wang Zepeng	2022	Low bias	Low bias	Bias ambiguity	Bias ambiguity	Low bias	Low bias	Low bias
Deng Guohua	2022	Low bias	Low bias	Bias ambiguity	Bias ambiguity	Low bias	Low bias	Low bias
Li Fangqing	2017	Low bias	Low bias	Bias ambiguity	Bias ambiguity	Low bias	Low bias	Low bias
Yu Changwei	2018	Low bias	Low bias	Bias ambiguity	Bias ambiguity	Low bias	Low bias	Low bias
Zhang Xi	2020	Low bias	Low bias	Bias ambiguity	Bias ambiguity	Low bias	Low bias	Low bias
Deng Bin	2020	Low bias	Low bias	Bias ambiguity	Bias ambiguity	Low bias	Low bias	Low bias
Kwon Young Sil	2020	Low bias	Low bias	Bias ambiguity	Bias ambiguity	Low bias	Low bias	Low bias
Zhang Aiping	2020	Low bias	Low bias	Bias ambiguity	Bias ambiguity	Low bias	Low bias	Low bias
Yang Xianzhou	2017	Low bias	Low bias	Bias ambiguity	Bias ambiguity	Low bias	Low bias	Low bias
Chen Rong	2018	Low bias	Low bias	Bias ambiguity	Bias ambiguity	Low bias	Low bias	Low bias
Yu Chunlei	2019	Low bias	Low bias	Bias ambiguity	Bias ambiguity	Low bias	Low bias	Low bias
Huang Minzhen	2018	Low bias	Low bias	Bias ambiguity	Bias ambiguity	Low bias	Low bias	Low bias
Yang Jie	2019	Bias ambiguity	Bias ambiguity	Bias ambiguity	Bias ambiguity	Low bias	Low bias	Bias ambiguity
Dong Dalong	2019	Low bias	Low bias	Bias ambiguity	Bias ambiguity	Low bias	Low bias	Low bias
Cheng Yan	2017	Low bias	Low bias	Bias ambiguity	Bias ambiguity	Low bias	Low bias	Low bias
Wei Nan Fu	2017	Low bias	Low bias	Bias ambiguity	Bias ambiguity	Low bias	Low bias	Low bias
Kateryna Bielka	2021	Low bias	Low bias	Low bias	Low bias	Low bias	Low bias	Low bias
Yan Tang	2023	Low bias	Low bias	Low bias	Low bias	Low bias	Low bias	Low bias
Liang Jin	2020	Low bias	Low bias	Bias ambiguity	Bias ambiguity	Low bias	Low bias	Bias ambiguity
Qiu Dongjie	2023	Low bias	Low bias	Low bias	Low bias	Low bias	Low bias	Low bias
Jianhong Hao	2019	Low bias	Low bias	Bias ambiguity	Bias ambiguity	Low bias	Low bias	Bias ambiguity

### 3.4 Results of index meta-analysis

#### 3.4.1 Blocking effective rate

**FIGURE 2:**
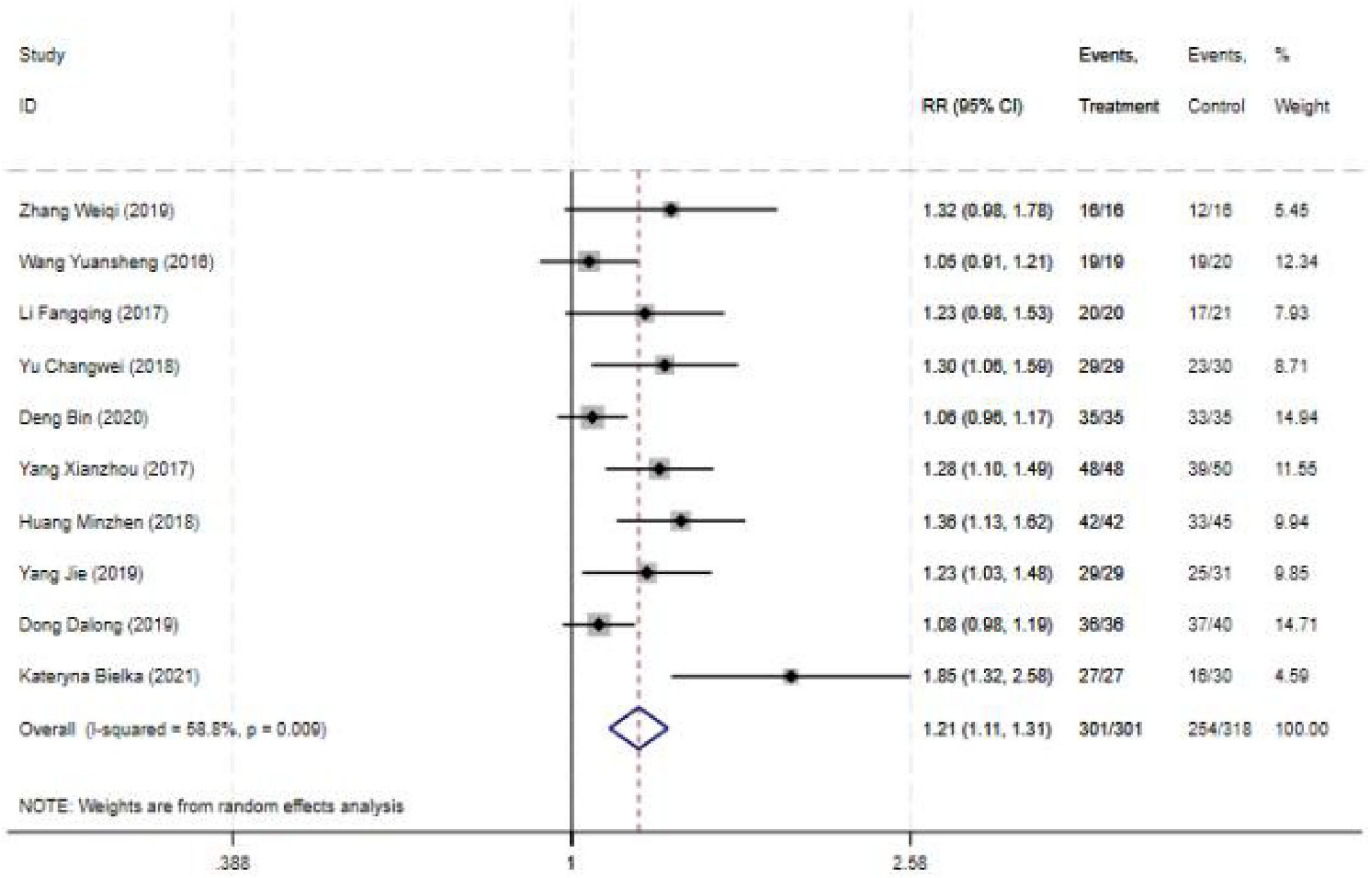
Forest plot comparing Blocking effective rate for ultrasound-guided nerve block vs. control group. RR: Relative risk; 95CI, 95% confidence intervals.

The blocking effective rate was reported in all 10 studies, and the results of an inter-study heterogeneity test were *P* < 0.001 and I^2^ = 58.8%. The meta-analysis using the random effects model showed that there was a statistically significant difference in the effective rate between the two groups [*RR* = 1.21 (1.11, 1.31), *P* < 0.001], and the effective rate of the experimental group was 1.21 times that of the control group (Figure 2 and Table 3).

**TABLE 3:** Effect size.

Index	Heterogeneity test		Pooled RR (95%CI)/SMD (95%CI)	*P*
	*P*	I^2^		
Blocking effective rate	0.01	58.80%	1.21(1.11, 1.31)	<0.01
Sensory nerve block onset time	<0.01	97.50%	−2.76(−3.67, −1.85)	<0.01
Sensory nerve block duration time	<0.01	94.40%	2.52(1.86, 3.18)	<0.01
Motor nerve block onset time	<0.01	96.40%	−1.94(−2.68, −1.19)	<0.01
Motor nerve block duration time	<0.01	97.60%	1.11(0.24, 1.98)	0.01
VAS score post operation 4H	<0.01	94.80%	−1.68(−2.68, −0.68)	<0.01
VAS score post operation 8H	<0.01	89.30%	−0.99(−1.66, −0.32)	<0.01
VAS score post operation 12H	<0.01	82.80%	−1.03(−1.48, −0.58)	<0.01
VAS score post operation 24H	<0.01	96.30%	−1.56(−2.36, −0.75)	<0.01
Adverse reaction rate	<0.01	74.40%	0.35(0.23, 0.55)	<0.01
Operating time	<0.01	92.80%	0.25(−0.36, 0.86)	0.43

#### 3.4.2 Sensory nerve block onset and duration time

**FIGURE 3:**
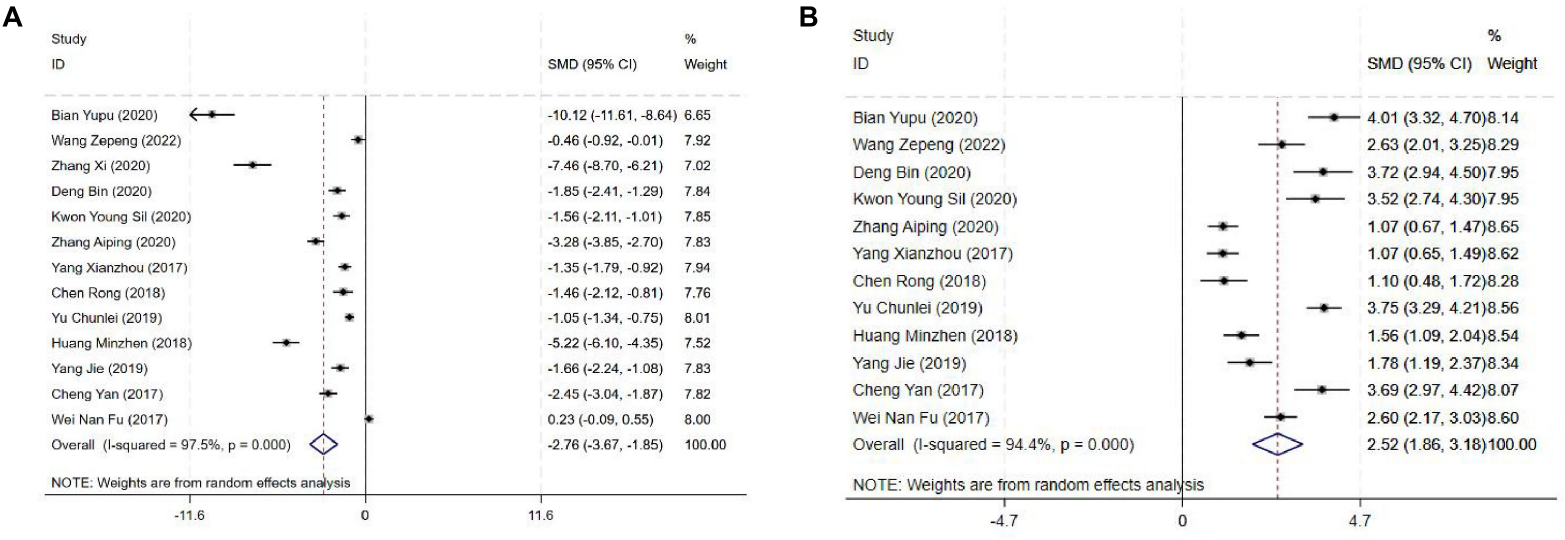
Forest plot comparing sensory nerve block. **(A)** Sensory nerve block onset time, **(B)** Sensory nerve block duration time. SMD, Standardized Mean Difference; 95%CI, 95% confidence intervals.

The block onset time was reported in 13 studies, and the results of an inter-study heterogeneity test were *P* < 0.001 and *I*^2^ = 97.5%. The meta-analysis using the random effects model showed that there was a statistically significant difference in sensory nerve block onset time between the two groups [SMD = −2.76 (−3.67, −1.85), *P* < 0.001], and the sensory nerve block onset time of the experimental group was shorter than that of the control group (Figure 3A and Table 3).

The block duration time was reported in 12 studies, and the results of an inter-study heterogeneity test were *P* < 0.001 and *I^2^* = 94.4%. A meta-analysis using a random effects model showed that sensory nerve block duration time difference between the two groups was statistically significant [SMD = 2.52(1.86,3.18), *P* < 0.01], and the sensory nerve block duration time of the experimental group was longer than that of the control group (Figure 3B and Table 3).

#### 3.4.3 Motor nerve block onset and duration time

**FIGURE 4:**
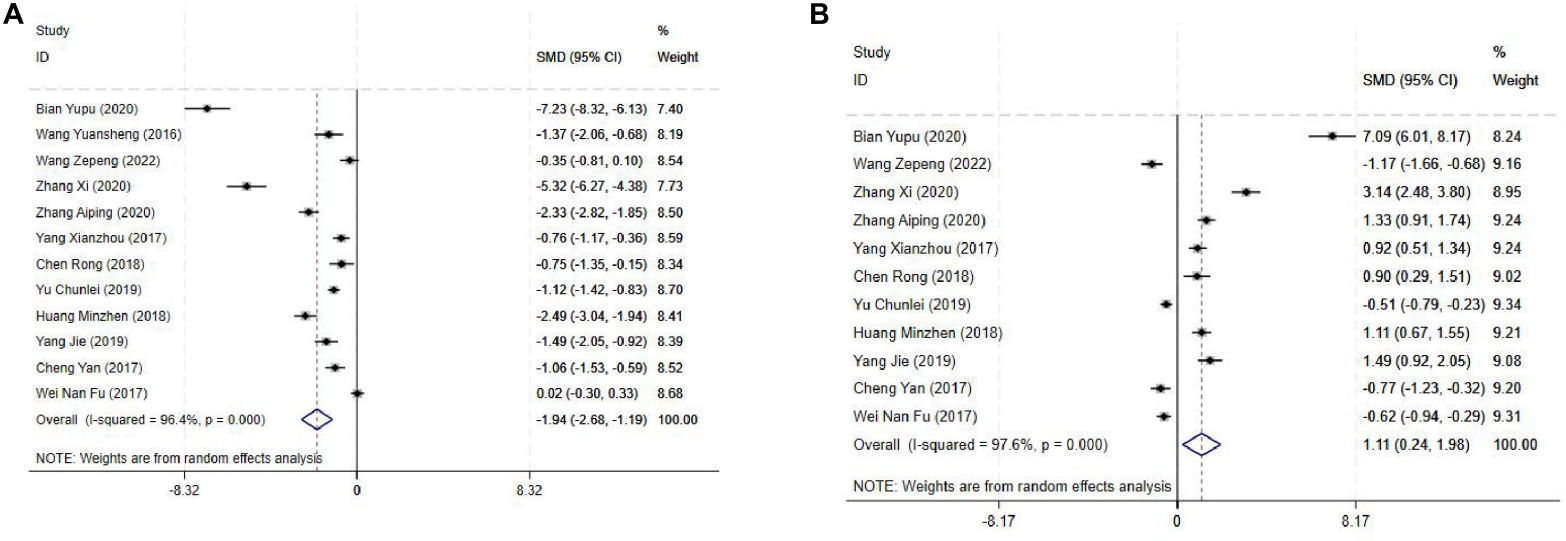
Forest plot comparing motor nerve block. **(A)** Motor nerve block onset time, **(B)** Motor nerve block duration time. SMD, Standardized Mean Difference; 95%CI, 95% confidence intervals.

The motor nerve block onset time was reported in 12 studies, and the results of an inter-study heterogeneity test were *P* < 0.001 and *I*^2^ = 96.4%. The meta-analysis using the random effects model showed that there was a statistically significant difference in motor nerve block onset time between the two groups [SMD = −1.94(−2.68, −1.19), *P* < 0.001], and the motor nerve block onset time of the experimental group was shorter than that of the control group (Figure 4A and Table 3).

The motor nerve block duration time was reported in 11 studies, and the results of an inter-study heterogeneity test were *P* < 0.001 and *I*^2^ = 97.6%. A meta-analysis using a random effects model showed that motor nerve block duration time difference between the two groups was statistically significant [SMD = 1.11(0.24,1.98), *P* = 0.01], and the motor nerve block duration time of the experimental group was longer than that of the control group (Figure 4B and Table 3).

#### 3.4.4 Analgesic effect

**FIGURE 5:**
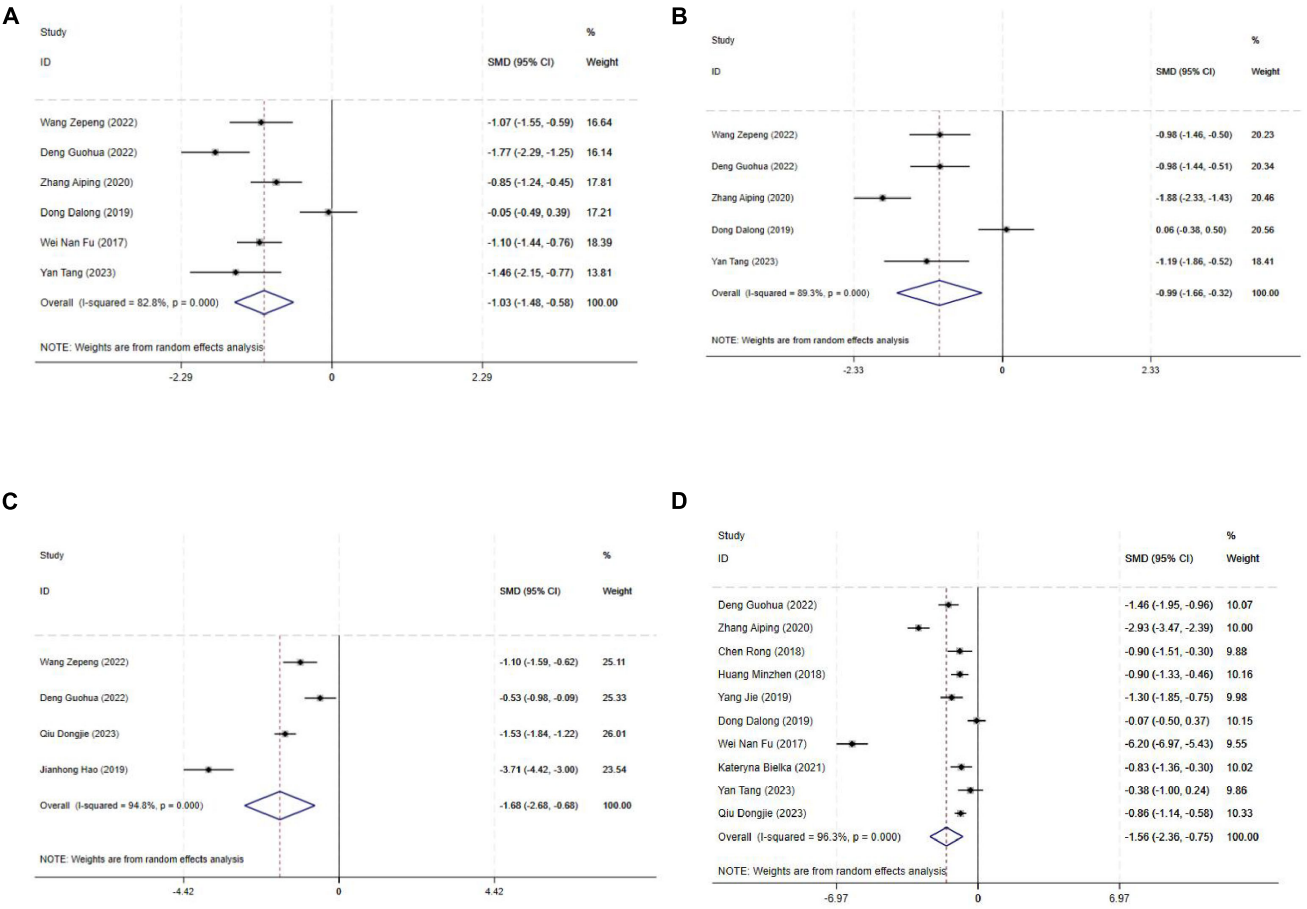
Forest plot comparing VAS score. **(A)** Post operation 4H, **(B)** post-operation 8H, **(C)** post-operation 12H, **(D)** post-operation 24H. SMD, Standardized Mean Difference; 95%CI, 95% confidence intervals.

The VAS score post operation 4H was reported in six studies, and the results of an inter-study heterogeneity test were *P* < 0.001 and *I^2^* = 94.80%. The meta-analysis using the random effects model showed that there was a statistically significant difference in the VAS score post operation 4H between the two groups [SMD = −1.68(−2.68, −0.68), *P* < 0.001] (Figure 5A and Table 3); The VAS score post operation 8H was reported in 5 studies, and the results of an inter-study heterogeneity test were *P* < 0.001 and *I*^2^ = 89.30%. The meta-analysis using the random effects model showed that there was a statistically significant difference the VAS score post operation 8H between the two groups [SMD = −0.99(−1.66, −0.32), *P* < 0.001] (Figure 5B and Table 3); The VAS score post operation 12H was reported in 4 studies, and the results of an inter-study heterogeneity test were *P* < 0.001 and *I*^2^ = 82.80%. The meta-analysis using the random effects model showed that there was a statistically significant difference in the VAS score post operation 12H between the two groups [SMD = −1.03(−1.48, −0.58), *P* < 0.001] (Figure 5C and Table 3); The VAS score post operation 24H was reported in 6 studies, and the results of an inter-study heterogeneity test were *P* < 0.001 and *I*^2^ = 96.30%. The meta-analysis using the random effects model showed that there was a statistically significant difference in the VAS score post operation 24H between the two groups [SMD = −1.56(−2.36, −0.75), *P* < 0.001] (Figure 5D and Table 3).

The VAS scores of the ultrasound-guided nerve blocks were lower than those of the control group at 4, 8, 12, and 24 h after surgery.

#### 3.4.5 Adverse reaction rate

**FIGURE 6:**
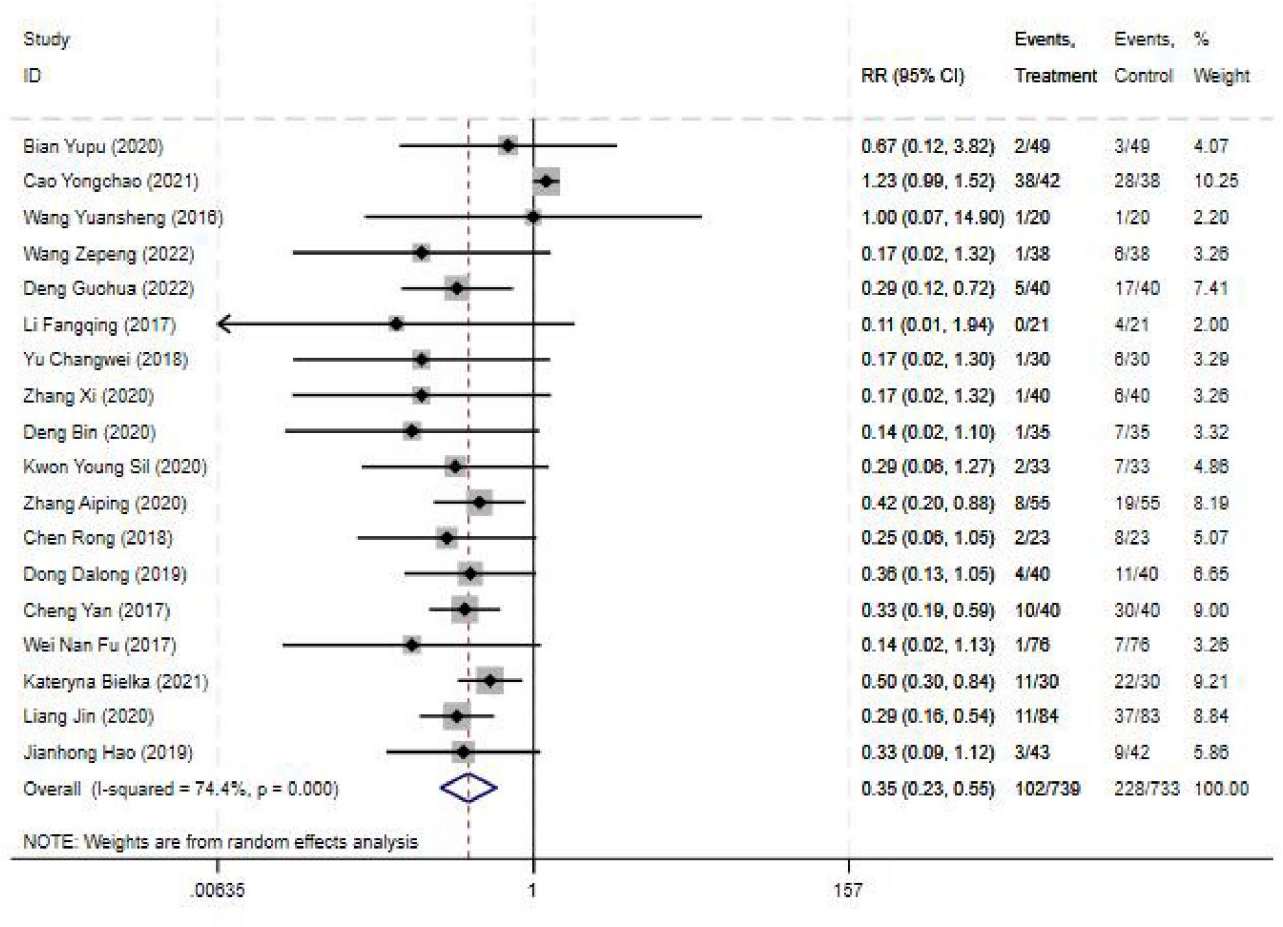
Forest plot comparing blocking effective rate for ultrasound-guided nerve block vs. control group. RR, Relative risk; 95%CI, 95% confidence intervals.

The Adverse reaction rate was reported in 18 studies, and the results of an inter-study heterogeneity test were *P* < 0.001 and *I*^2^ = 74.40%. The meta-analysis using the random effects model showed that there was a statistically significant difference in the adverse reaction rate between the two groups [RR = 0.35(0.23,0.55), *P* < 0.001], and the adverse reaction rate of the experimental group was 0.35 times that of the control group (Figure 6 and Table 3).

#### 3.4.6 Operating time

**FIGURE 7:**
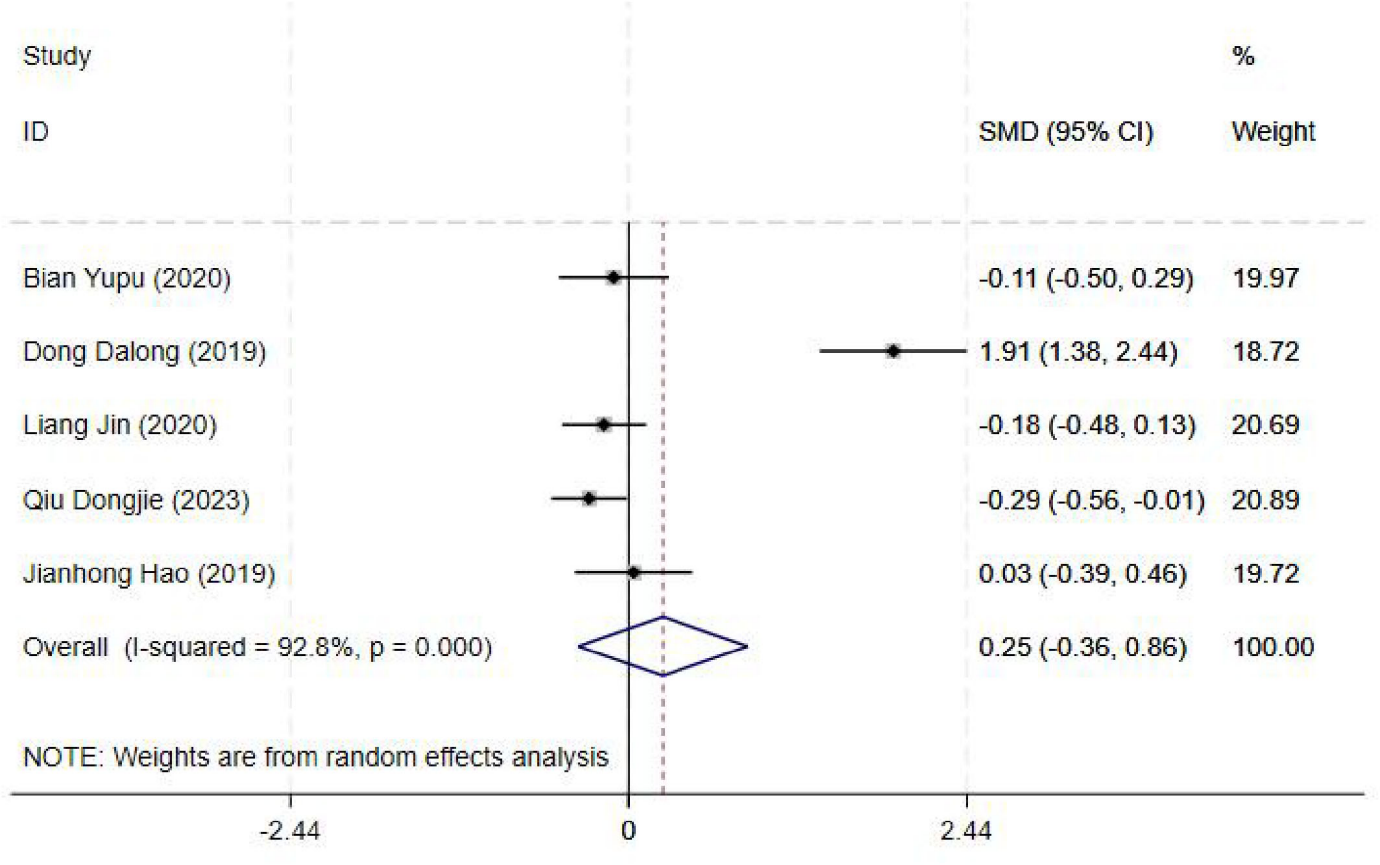
Forest plot comparing operating time for ultrasound-guided nerve block vs. control group. SMD, Standardized Mean Difference; 95%CI, 95% confidence intervals.

The operating time was reported in five studies, and the results of an inter-study heterogeneity test were *P* < 0.001 and *I*^2^ = 92.80%. The meta-analysis using the random effects model showed that there was no significant difference in operating time between the two groups [SMD = 0.25(−0.36, 0.86), *P* = 0.43] (Figure 7 and Table 3).

### 3.5 Publication bias

The Begg’s test results (Table 4) for the Blocking effective rate (*z* = −1.53, *p* = 0.13), Sensory nerve block onset time (*z* = −3.48, *p* = 2.00), Sensory nerve block duration time (*z* = 1.71, *p* = 0.09), Motor nerve block onset time (*z* = −2.40, *p* = 1.98), Motor nerve block duration time (*z* = 2.02, *p* = 0.04), VAS score post operation 4H (*z* = −1.02, *p* = 1.69), VAS score post operation 8H (*z* = −0.73, *p* = 1.79), VAS score post operation 12H (*z* = −1.13, *p* = 1.74), VAS score post operation 24H (*z* = −1.25, *p* = 0.13), Adverse reaction rate (*z* = −0.68, *p* = 1.50), Operating time (*z* = *2.20, p* = 0.03) and the scatter distribution of each study was basically symmetrical (Supplementary Figures 1–10 in Supplementary File 2).

**TABLE 4:** Publication bias results of Begg’s test.

Index	Begg’s test	
	*Z*	*P*
Blocking effective rate	−1.53	0.13
Sensory nerve block onset time	−3.48	2.00
Sensory nerve block duration time	1.71	0.09
Motor nerve block onset time	−2.40	1.98
Motor nerve block duration time	2.02	0.04
VAS score post operation 4H	−1.02	1.69
VAS score post operation 8H	−0.73	1.54
VAS score post operation 12H	−1.13	1.74
VAS score post operation 24H	−1.25	1.79
Adverse reaction rate	−0.68	1.50
Operating time	2.20	0.03

The results indicated that there was no publication bias except motor nerve block duration time, Operating time, and then the two indexes used the scissoring method. But the number of references was not filled after the scissoring method. So there may be a publication bias in the two indexes, the results of the meta-analysis of the two indexes should be interpreted with caution.

### 3.6 Sensitivity analysis

After deleting all studies one by one, the obtained 95% CI did not cross the invalid line, and the point of combined effect size was still within the original 95% CI, indicating that the results of this study were relatively robust (Supplementary Tables 1–11 in Supplementary File 2).

## 4 Discussion

The aging process is accompanied by the emergence of a diverse spectrum of both acute and chronic illnesses. This physiological decline manifests in a gradual deterioration of bodily functions and a diminishing organ reserve capacity, rendering elderly individuals less tolerant of surgical trauma. These factors present significant challenges to the advancement of surgical interventions in this patient population (46). These characteristics can significantly complicate upper limb, lower limb, abdominal, and thoracic surgical procedures. For instance, patients with hypertension exhibit blood pressure fluctuations during surgery, resulting in decreased vascular wall elasticity and increased fragility. This heightened susceptibility can readily lead to serious adverse events, such as cerebrovascular accidents and myocardial infarction (47, 48). To enhance the safety of surgical interventions, more nuanced anesthetic strategies are imperative. Scholars have demonstrated that in upper limb surgery, brachial plexus block can accurately block nerve conduction, effectively reduce the pain in the surgical area, reduce the number of general anesthesia drugs, and reduce the impact on cardiovascular, respiratory, and other systems, thus reducing the risk of surgery (49, 50). Therefore, it emerges as a crucial component in optimizing patient recovery and ensuring the quality of postoperative care.

This study undertook a rigorous systematic review and meta-analysis to comparatively assess the efficacy and safety of ultrasound-guided nerve block versus other anesthetic modalities in geriatric surgical patients. The ultrasound-guided nerve block technique exhibited a superior block success rate. Notably, the onset of sensory nerve block was more rapid in the experimental cohort compared to the control group, while the duration of sensory nerve block was prolonged. These findings underscore the unique advantages of ultrasound-guided nerve block in augmenting surgical safety and enhancing patient comfort within the clinical setting. This meta-analysis provides a novel perspective on the comparative evaluation of ultrasound-guided nerve block and alternative anesthetic approaches. Through a meticulous examination of key indicators, including block success rate, onset latency, analgesic duration, and complication incidence, this study offers a comprehensive assessment of these anesthetic techniques within the geriatric surgical patient population. The study findings emphasize the pivotal role of ultrasound-guided nerve blocks in contemporary clinical practice, particularly their exceptional efficacy in mitigating postoperative wound pain and enhancing surgical safety.

A meta-analysis of diverse efficacy indicators revealed that ultrasound-guided nerve blocks exhibit numerous advantages. Notably, the experimental group demonstrated superior block efficacy compared to the control group. This enhancement can be attributed to the inherent capacity of ultrasound imaging to delineate the intricate anatomical relationships between nerves and surrounding tissues. This real-time visualization enables anesthesiologists to precisely target the nerves for drug injection, thereby optimizing the blockade of neural conduction. Yu et al. (51) found that ultrasound-guided subsheath sciatic nerve block was more effective than rapidly and completely achieving tibial and foot surgical sensory block, with faster start time and comparable safety.

A noteworthy observation pertains to the relevant indicators of sensory and motor nerve blockade. Notably, the latency to onset of the block in the experimental cohort was significantly reduced compared to the control group, while the duration of the block was markedly prolonged. Consistent with these findings, Haley et al. (9) reported shorter onset times for sensory nerve block in a study of ultrasound-guided suprazygomatic nerve block to the pterygopalatine fossa compared to other anesthetic techniques. This rapid onset of anesthesia can expedite the provision of adequate pain control at the commencement of surgery, thereby mitigating patient discomfort. This finding carries significant implications for the peri-operative management of elderly surgical patients. Furthermore, a randomized, double-blind, placebo-controlled trial conducted by Rambhia et al. (52) investigating ultrasound-guided ganglion block following total knee arthroplasty demonstrated that prolonged block duration not only facilitated continuous postoperative analgesia but also mitigated the risk of various complications arising from pain-induced physiological stress responses, thereby accelerating patient recovery. Their findings align with the conclusions drawn from this study.

Analysis of the Visual Analog Scale (VAS) scores at various time points postoperatively revealed that the ultrasound-guided nerve block group exhibited significantly lower VAS scores at 4, 8, 12, and 24 h compared to the control group. This finding strongly suggests that this technique may confer more efficacious postoperative analgesia in elderly surgical patients. A study published in the American Journal of Emergency Medicine (53) corroborated these findings by comparing the analgesic efficacy of ultrasound-guided femoral nerve block (USFNB) in patients with both intracapsular and extracapsular hip fractures. This evidence underscores the critical importance of effective analgesia, particularly in geriatric patients. Adequate pain management mitigates the physiological stress response elicited by pain, thereby promoting improved sleep quality, dietary intake, and functional recovery.

Furthermore, regarding the incidence of adverse events, the experimental group exhibited a significantly lower rate, amounting to only 0.35 times that observed in the control group. This finding unequivocally demonstrates the enhanced safety profile of ultrasound-guided nerve block. This may be due to the real-time visual guidance of ultrasound. Bowness (54) posited that meticulous adjustment of image display parameters, including focus, depth, and gain (brightness), can effectively mitigate the risk of inadvertent injury to vital surrounding structures, such as blood vessels and nerves, during needle puncture. This optimization of image visualization is crucial in minimizing the likelihood of adverse events stemming from procedural errors.

This study, however, also identified several limitations. Notably, most heterogeneity tests revealed substantial heterogeneity among studies, likely attributable to variations in patient characteristics (e.g., age, comorbidities, physical condition), surgical procedures, anesthetic choices, ultrasound equipment, and operator expertise. The inconsistency of these factors may have introduced bias into the study results and limited the generalizability of the findings. Publication bias analysis indicated the absence of bias for most outcomes, with the notable exception of motor nerve block duration and operative time, which exhibited evidence of potential publication bias. Sensitivity analyses demonstrated that the study results were generally robust and reliable, with minimal influence from individual studies.

This study underscores the substantial advantages of ultrasound-guided nerve block in terms of both efficacy and safety within the elderly surgical population. However, recognizing the inherent heterogeneity of the existing evidence base and the potential for publication bias, further investigation is warranted. Specifically, larger, multi-center trials with rigorous methodology are required to elucidate the optimal application paradigm for ultrasound-guided nerve block in this patient cohort. Key areas for further exploration include: (1) the identification of appropriate patient selection criteria, (2) the refinement of operational protocols, and (3) the judicious selection of analgesic agents and their appropriate dosing regimens. These endeavors will contribute to a more robust evidence base to guide clinical practice.

### 4.1 Study limitation

While the scope of the literature search was not explicitly limited, the inclusion of publications in languages other than English was constrained by language barriers. Consequently, the included literature predominantly comprised Chinese publications, followed by English, raising concerns regarding potential selection bias. Furthermore, the possibility of publication bias cannot be entirely ruled out, as the search may not have encompassed all available gray literature, such as conference proceedings and supplements. Among the included studies, methodological limitations exist, particularly concerning the presence of random and blinded procedures, which significantly impact the overall quality of the evidence. The need for robust, high-quality evidence remains, specifically large-scale, multi-center, randomized controlled trials employing optimal study designs to investigate the efficacy and safety of ultrasound-guided nerve blocks in elderly surgical patients.

## 5 Conclusion

This meta-analysis provides a comprehensive exploration of ultrasound-guided nerve block utilization in elderly surgical patients, offering a nuanced understanding of its clinical significance. Our findings unequivocally demonstrate that ultrasound-guided nerve block confers substantial advantages. In terms of efficacy, it exhibits a superior block success rate, more rapid onset times for both sensory and motor nerve blockade, and prolonged block duration. These factors translate into efficient pain management during and after surgery, as evidenced by the lower VAS scores at multiple time points post-operation. This not only enhances patient comfort but also significantly mitigates the stress response associated with pain, thereby facilitating improved recovery trajectories in terms of sleep quality, dietary intake, and functional restoration.

## Data Availability

The data analyzed in this study is subject to the following licenses/restrictions: Heterogeneity of Studies: The included studies in the meta-analysis may have significant differences in terms of study design (e.g., randomized controlled trials vs. observational studies), patient populations (varying ages, comorbidities, and types of surgeries among elderly patients), the specific techniques of ultrasound-guided nerve blocks used (different approaches, frequencies, etc.), and outcome measures. This heterogeneity can limit the generalizability of the pooled results and may introduce bias. Requests to access these datasets should be directed to wangrong8710@163.com.
