# Supplementary material for: Efficacy and safety of ultrasound-guided nerve blocks in elderly surgical patients: a meta-analysis
Source: Front Med (Lausanne). 2025 Aug 18;12:1580172. doi: 10.3389/fmed.2025.1580172 (PMC12399598; doi:10.3389/fmed.2025.1580172)
Supplement: Supplementary file 1 [file Supplementary_file_1.docx]

| **Supplementary file 1 Table 1 Search strategy** | | |
| --- | --- | --- |
| Database | Search strategy | Search result |
| Pubmed | (((((((((nerve block[MeSH Major Topic]) OR (Ultrasound-guided nerve block[Title/Abstract])) OR (US-guided SNB[Title/Abstract])) OR (nerve block[Title/Abstract])) OR (Ultrasound-guided scalp nerve block[Title/Abstract])) OR (Block, Nerve[Title/Abstract])) OR (Nerve Blocks[Title/Abstract])) OR (Blockade, Nerve[Title/Abstract])) AND (((((((((((((Elderly patients[Title/Abstract]) OR (old people[Title/Abstract])) OR (geriatrics[Title/Abstract])) OR (geriatrist[Title/Abstract])) OR (Geriatric Anesthesia[Title/Abstract])) OR (Anesthesias, Geriatric[Title/Abstract])) OR (Geriatric Anesthesias[Title/Abstract])) OR (Anesthesia, Geriatric[Title/Abstract])) OR (Anesthesia, Geriatrics[Title/Abstract])) OR (Anesthesias, Geriatrics[Title/Abstract])) OR (Geriatrics Anesthesia[Title/Abstract])) OR (Geriatrics Anesthesias[Title/Abstract])) OR (Geriatric Anesthesia[MeSH Major Topic])) | 407 |
| Web of sicience | #1 ((((((((TS=(nerve block)) OR AB=(Ultrasound-guided nerve block)) OR AB=(US-guided SNB)) OR AB=(nerve block)) OR AB=(Ultrasound-guided scalp nerve block)) OR AB=(Block, Nerve)) OR AB=(Blocks, Nerve)) OR AB=(Nerve Blocks)) OR AB=(Blockade, Nerve)  #2 ((((((((((((TS=(Geriatric Anesthesia)) OR AB=(Elderly patients)) OR AB=(old people)) OR AB=(geriatrics)) OR AB=(geriatrist)) OR AB=(Geriatric Anesthesia)) OR AB=(Anesthesias, Geriatric)) OR AB=(Geriatric Anesthesias)) OR AB=(Anesthesia, Geriatric)) OR AB=(Anesthesia, Geriatrics)) OR AB=(Anesthesias, Geriatrics)) OR AB=(Geriatrics Anesthesia)) OR AB=(Geriatrics Anesthesias) (#1) AND (#2) | 332 |
| Cochrane Library | (ultrasound-guided nerve block) AND (elderly patients) AND (RCT) | 2 |
| Embase | #1：('ultrasound-guided nerve block' OR ('ultrasound guided' AND ('nerve'/exp OR nerve) AND block) OR 'nerve block'/exp OR 'nerve block' OR (('nerve'/exp OR nerve) AND block) OR 'us-guided snb' OR ('us guided' AND snb) OR 'ultrasound-guided scalp nerve block' OR ('ultrasound guided' AND ('scalp'/exp OR scalp) AND ('nerve'/exp OR nerve) AND block)) AND ('nerve nerve blocks blockade' OR (('nerve'/exp OR nerve) AND blocks AND blockade)) #2:'elderly patients' OR (('elderly'/exp OR elderly) AND ('patients'/exp OR patients)) OR 'old people' OR (old AND people) OR 'geriatrics'/exp OR geriatrics OR geriatrist OR 'geriatric anesthesia anesthesias' OR (('geriatric'/exp OR geriatric) AND ('anesthesia'/exp OR anesthesia) AND anesthesias) OR 'geriatrics anesthesias' OR (('geriatrics'/exp OR geriatrics) AND anesthesias) | 483 |
| CNKI | (SU%='超声引导下神经阻滞'+'超声引导神经阻滞') AND (SU%='老年麻醉'+'老年患者'+'老年病人麻醉'+'老年病人') | 871 |
| Wanfang | （（中英文扩展&主题词扩展）：全部:(超声引导下神经阻滞\+超声引导神经阻滞\+神经阻滞\+超声引导头皮神经阻滞) and 全部:(老年麻醉\+老年患者\+老年病人麻醉\+老年病人) | 0 |
| VIP | (((题名=超声引导下神经阻滞 OR 题名=超声引导神经阻滞) OR 题名=神经阻滞) OR 题名=超声引导头皮神经阻滞)) AND (((题名=老年麻醉 OR 题名=老年患者) OR 题名=老年病人麻醉) OR 题名=老年病人)) | 492 |
| Total |  | 2587 |
| **Note: The literature was searched until January 2, 2025** | |  |

| **Supplementary file 1 Table 2** | | | | | | | | | |
| --- | --- | --- | --- | --- | --- | --- | --- | --- | --- |
| **First author** | **Year** | **Research design method** | **locations** | | **specific treatment steps** | | **Adverse reactions** | **Inclusion criteria** | **Exclusion criteria** |
|  |  |  | **Exp.** | **Con.** | **Exp.** | **Con.** |  |  |  |
| **Zhang Weiqi** | **2019** | **Retrospective study** | From the center of the spine to the transverse process | From the center of the spine to the transverse process | Local infiltration anesthesia was performed with 1% lidocaine, and dural puncture was performed with ultrasound guided technique. The puncture was also performed at the center of the patient's spine. When the needle tip reached the nerve at the transverse process, the patient was injected with a concentration of 0.5% and a dose of 20 ml ropivacaine. The sciatic nerve outside the semitendinosus muscle and biceps femoris of the patient will form triangle or oval-shaped hyperecho. The needle will be punctured next to the sciatic nerve by ultrasound guidance technology, and then the patient will be injected with ropivacaine with a concentration of 0.5% and a dose of 25 ml (Manufacturer: Chengdu Tiantaishan Pharmaceutical Co., LTD.; Approval number: H20052666) 5 mL, after injection to observe the patient's condition, ephedrine hydrochloride tablets can be used if necessary (manufacturer: Dandong Yichuang Chinese Medicine Co., LTD.; Approval number: Sinopharm Approval number H21022178) Maintain blood pressure. | Local infiltration anesthesia was performed around the iliac ridge of the patient with 1% lidocaine as an anesthetic. External puncture was performed in the epidural area, and vertical puncture was performed at the exact center of the spine about 5 cm. The needle was slightly pulled out at the transverse process, and then slowly inclined upward to insert the needle 0.5 cm at the upper end of the transverse process | not clear | NA | NA |
| **Bian Yupu** | **2020** | **Retrospective study** | Location of the lumbar plexus nerve and sciatic nerve | Epidural space | Ultrasound-guided low back plexus-sciatic nerve block anesthesia was used: The patient was placed in lateral kneeling position, and the lumbar plexus and sciatic nerve were punctured by ultrasound. After repeated positioning, 5 mL of 0.5% ropivacaine was injected into each puncture site, and 25 mL of 0.5% ropivacaine was injected again when blood return was not detected under ultrasound. | The patient was instructed to take the lateral position and select the puncture point 1.5 cm from the median line as the puncture point. The puncture needle was inserted into the median line at 75° between the skin and the skin. After entering the epidural space through the subcutaneous, erector spinal muscle and ligamenta flava, the catheter passed through the needle, withdrew the puncture needle and fixed the catheter. After changing the patient's position, 0.5% ropivacaine hydrochloride for injection was injected through the catheter | Dyspnea, Nerve injury, Urinary retention | ① The first artificial knee replacement; ② Age ≥65 years old; ③ It meets the relevant diagnostic criteria of traumatic arthritis, rheumatoid arthritis and osteoarthritis. | ① Serious dysfunction of heart, kidney and other organs; ② There are contraindications of knee arthroplasty; ③ Coagulation dysfunction; ④ suffers from mental illness; (5) Allergies to narcotic drugs related to this study. |
| **Cao Yongchao** | **2021** | **Retrospective study** | The middle oblique muscle is located between the upper, middle and lower trunk of the brachial plexus | Above the hyoid bone, within the intermuscular groove of the anterior middle oblique muscle | After routine monitoring, intravenous access was opened, oxygen was given, midazolam 1mg, Sufentanil 5μg sedation and analgesia were given 5min before the start. The ultrasound probe uses a disposable sterile protective sleeve and is then anesthetized under ultrasound guidance. The linear array high-frequency ultrasound probe was placed 2cm above the clavicle to scan up and down. When the images of the upper, middle and lower branches of the brachial plexus nerve were clearly visible, the puncture needle was inserted from the outside, and the in-plane technique was used to Pierce the puncture needle between the middle scalenes muscle and the upper, middle and lower branches of the brachial plexus. A small amount of 0.375% ropivacaine was injected for 20m multiple times | Conventional brachial plexus block anesthesia was used. The puncture site was selected above the thyrohyoid muscle and in the intermuscular groove of the anterior middle scalene muscle, and puncture was performed with a 7-gauge needle. About 1.5cm was inserted and the needle was fixed. If there was no bleeding after withdrawal, 20ml of 0.375% ropivacaine (manufacturer: Qilu Pharmaceutical Co., LTD., approval number: H20052690) was injected slowly to complete anesthesia | Hematoma, Pneumothorax, Nausea, Vomiting | (1) not less than 60 years old, not more than 80 years old; (2) Having indications related to upper extremity orthopedic surgery; (3) Patient data are clear and complete, and informed consent is signed | (1) Abnormal vital organs, such as brain, heart, kidney, etc.; (2) Allergic to the drug used; (3) Diseases or abnormalities of the immune system or nervous system; (4) combined with coagulation dysfunction; (5) There are major infectious diseases. |
| **Wang Yuansheng** | **2016** | **Retrospective study** | The middle of the anterior middle oblique muscle | The horizontal intersection line between the intermuscular sulcus and the annular cartilage | Ultrasound locator (ALOKA, 13) was used to scan the brachial plexus nerve of the intermuscular groove (frequency 5-10 MHz). The hypoechoic target nerve trunk was found in the middle of the anterior middle scalenus muscle. The puncture needle was inserted into the lateral plane of the ultrasound probe for nerve block, and 30 ml 0.5% ropivacaine was injected into the target nerve after reaching the target nerve (AstraZeneca AB, 100 mg / 10ml, registration number: H20140763), adjust the direction and depth of the puncture needle during injection so that the target nerve is wrapped by local anesthetic as much as possible | With the aid of nerve stimulation instrument, the horizontal cross line between the intermuscular groove and the cartilage of the annulus was taken as the puncture point, and Stimuplex 2DIG(Braun, USA) nerve stimulation instrument was used. The initial current was 1 mA, and when the stimulation needle induced muscle contraction of the upper limb, the current was reduced to 0.3 ~ 0.5 mA. If there was still muscle fibrillation, the positioning was considered accurate. After withdrawal without blood and cerebrospinal fluid, 30 ml 0.5% ropivacaine was injected. If the target nerve cannot be located, administer the drug to the transverse process of the sixth cervical vertebra. The sensory and motor blocks in the ulnar nerve, radial nerve and median nerve innervation were evaluated by acupuncture method and modified Bromage scoring method 1, 3, 5, 10, 15, 20, 25 and 30 min after the puncture block was completed. | Hematoma, Block failure | ≥65 years of age,  ASAI~ Class II; | Preoperative complications were neurological diseases, hepatic and renal insufficiency, brachial plexus injury, coagulation dysfunction, local anesthetic allergy, puncture site infection and damage |
| **Wang Zepeng** | **2022** | **Retrospective study** | At the groin | Intervertebral Spaces of L3 to L4 | Femoral nerve block and lateral femoral cutaneous nerve block were used in the group. The patient was supine and the affected limb was rotated out appropriately. The ultrasonic probe was placed in the patient's groin, and the femoral nerve of the patient was located and properly labeled by the ultrasonic instrument. The frequency of the ultrasonic probe was (6-13) MHz, the needle insertion point was defined, and the needle was vertically inserted with the help of ultrasound. After blood was drawn back, 0.5% ropivacaine (10 mL) was injected into the upper and lower surfaces of the femoral nerve successively. The lateral femoral cutaneous nerve was identified by moving the ultrasound probe appropriately, and the intraplane puncture was performed, and 0.5% ropivacaine (3 mL) was injected. Knee anesthesia test was performed after 20 min, and surgery could be performed after no abnormality, and analgesia pump was properly connected for postoperative analgesia. | Combined lumbar and epidural anesthesia was performed. The patient was maintained in the lateral position, the affected limb was rotated externally, and puncture was performed in the L3 ~ L4 vertebral space of the patient. After the epidural puncture was successful, the lumbar puncture needle was inserted into the epidural puncture needle, the arachnoid was punctured, the needle core was slowly pulled out, and the outflow of cerebrospinal fluid was observed. 0.5% bupivacaine (10 m g) was injected, the lumbar puncture needle was withdrawn, and an epidural catheter was placed. After observation for 5 minutes, no high blocking plane was found, and the operation could be started. Combined with the intraoperative situation, local anesthesia was administered through an epidural catheter to maintain intraoperative anesthesia | Urinary retention, Headache, Nausea and vomiting | ① Patellar fracture confirmed by clinical CT or MRI imaging; ② The age of the patient is over 18 years old and the gender is not limited; ③ The time between fracture occurrence and operation is not more than 7 days; ④ fracture displacement more than 3 m m; ⑤ Patients and their families sign informed agreements | ① There is a history of patellar fracture; ② Orthopedic disease or pathological fracture affecting knee joint function before fracture; (3) Patients with mental diseases, skin infections, and coagulation disorders; (4) Surgical contraindications or intolerances. |
| **Deng Guohua** | **2022** | **Retrospective study** | Two cm below the inguinal ligament | Connect the self-controlled analgesic pump to the peripheral venous access | After applying a femoral nerve block, the probe was placed 2 cm below the patient's inguinal ligament using the DW-PF520 color ultrasound guide system. The long axis of the probe was perpendicular to the femur, and the probe was moved outward so that the iliac fascia between the sartor and iliopsoas muscles could be clearly detected. Then an ultrasound beam was scanned from the lateral thigh at a surface of 35~45° to the skin. An 18G trocar was used and injected into the iliac fascia space from the sartor muscle. After confirming that there was no blood return, 10 mL 0.2% ropivacaine (national drug approval number H20060137) was injected. During the injection, the puncture needle was inserted from deep within the iliac fascia to the femoral nerve, and then 10 mL normal saline was injected to expand the femoral nerve space under the iliac fascia. The epidural catheter was placed in the iliac fascia space near the femoral nerve, and 3-5 mL normal saline was injected to observe the diffusion effect. If it spreads around the nerve, a catheter can be placed and a disposable infusion pump connected. The drug in the infusion pump was 100 mL 0.2% ropivacaine. The background flow rate was set to 2 mL/h, patient-controlled analgesia was set to 0.5 mL, and the locking time was set to 15 min. | The patient was injected intravenously at the end of the operation, and the drug was dezocine (National drug approval number H20080329), the dose was 5 mg. Connect the self-controlled analgesia pump to the peripheral venous access, and inject 100 mL of sufentanil (National drug approval number H20054171) 4µg /kg solution. The background flow rate was 2 mL/h, patient-controlled analgesia was 0.5 mL, and the locking time was 15 min | Delirium, Urinary Retention, Nausea and Vomiting, Cognitive Dysfunction | ① Both groups met the indications of knee replacement; ② All patients underwent unilateral knee joint replacement; ③ None of the patients had mental abnormalities or family genetic history; ④ The patient's medical history is complete; ⑤ Patients and family members are informed and sign consent forms | ① There are severe tumors; ② Traumatic arthritis, class  Rheumatoid arthritis, coagulation disorders; ③ Patients with a history of knee surgery and mental illness; ④ lower extremity nerve injury; ⑤ can not cooperate with postoperative functional exercise. |
| **Li Fangqing** | **2017** | **Retrospective study** | At the intermuscular groove nerve tissue | The anterior and middle scalene muscles are located in the intermuscular grooves and above the scapular-hyoid muscles | Ultrasound diagnostic instrument was used for localization, and the nerves and surrounding tissues were scanned under ultrasound guidance to locate the blocked site of the patient. The depth and Angle of the needle were adjusted according to the ultrasound image. Under ultrasound guidance, the needle was inserted into the nerve tissue of the intermuscular sulci, and 20ml0.375% L-bupivacaine +1% lidocaine was injected after blood and gas were removed | Puncture was performed in the intermuscular sulci of the anterior and middle scalene muscles and above the omohyoid muscle. A 7-gauge injection needle was used to insert the needle into the skin at a height of 3cm to 4cm above the clavicle and 2cm to 3cm vertically. During the injection process, there was a strange sensation or a sense of breaking through the sheath, and no blood or gas was extracted. After pushing without resistance, 20ml0.375% L-bupivacaine +1% lidocaine was injected | Accidental vascular puncture,Nerve injury | NA | ① Patients with coagulation dysfunction; ② Lactation or pregnancy patients; ③ Patients with dysfunction of vital organs such as heart, brain, liver and kidney; ④ Mental patients; ⑤ Patients with drug allergy history |
| **Yu Changwei** | **2018** | **Retrospective study** | The superior and middle trunk of the brachial plexus nerve between the anterior oblique muscle and the middle oblique muscle, located above and behind the subclavian artery of the brachial plexus | Insert the needle between the anterior oblique muscle and the middle oblique muscle, 1cm above the midpoint of the clavicle | The patient's neck brachial plexus was scanned by Sonosite portable ultrasound system. The 6-12 Hz high-frequency linear array probe was first placed in the center of the neck, and the thyroid gland was observed. The probe moved laterally to reveal the internal jugular vein and the common carotid artery. The upper and middle branches of the low-echo round or oval brachial plexus nerve were identified in the middle of the anterior scalenes and the middle scalenes muscle. The ultrasound probe was moved gently to ensure that the images of the two branches of the brachial plexus nerve were located in the center of the ultrasound image. The extension tube was connected to the syringe, and the puncture needle was inserted from the lateral end of the ultrasound probe. The depth and Angle of injection were adjusted under the display of ultrasound images (see Figure 1), and 0.375% ropivacaine 10 mL was injected from the middle trunk of the brachial plexus slowly to the brachial plexus nerve after the withdrawal was confirmed to be blood-free [4-5], and the local anesthetic could be observed to spread around the brachial plexus through ultrasound (see Figure 2). After the needle withdrawal, the probe was moved towards the clavicle, and a honeycomb low echo was observed in the supraclaricular fossa, which was the brachial plexus, as well as structures such as subclavian artery, parietal pleura, and ribs (see Figure 3). The puncture needle was inserted from the lateral end of the ultrasound probe, and was placed around the upper brachial plexus behind the subclavian artery under the guidance of ultrasound using the in-plane technique. 0.375% ropivacaine 10 mL was injected after blood was withdrawn. Ultrasound shows a local anesthetic wrapped around the brachial plexus | According to the anatomical location, the needle was inserted between the anterior scalene muscle and the middle scalene muscle, and the blind probe was made into the intermuscular sulcus until the patient complained of different sensations. After no blood was drawn back, 0.375% ropivacaine was injected 10 mL. After the needle was withdrawn, the puncture needle was inserted about 1 cm above the midpoint of the clavicle in the backward, internal and downward directions to find the first rib, and the rib could be punctured by inserting the needle 1 ~ 3 cm. 10 mL 0.375% ropivacaine was fanned along the ribs. During the operation, 5 ~ 10 mg of dezocine and/or 5 μg of sufentanil were given intravenously to patients with incomplete analgesic effect. Patients with obvious pain intolerance were replaced by laryngeal mask implantation with general anesthesia | Vascular puncture, Pneumothorax, Horner's syndrome | NA | NA |
| **Zhang Xi** | **2020** | **Retrospective study** | Puncture 4 to 5 cm beside the L4 spinous process | Puncture is performed in the L2-3 intervertebral space, entering the dural cavity and then inserting a lumbar puncture needle | Use ultrasound-guided nerve block anesthesia: After entering the operating room, routine ECG monitoring was performed, blood pressure, pulse, blood oxygen and ECG were measured, oxygen inhalation was given to the mask, and the patient was assisted in the lateral and lateral lateral position. Sufentanil 10 μg and midazolium 1 mg were first injected intravenously for sedation. The ultrasound probe was placed on the iliac bone, and the quadrate and psoas major muscles were clearly displayed. The vertical muscle and L4 transverse process were punctured 4-5 cm next to the L4 spinous process, the tip of the needle to the nerve root, and withdrawal was performed. If there was no blood and cerebrospinal fluid, 25 ml of ropivacaine with a concentration of 0.5% was slowly injected. Then the ultrasound swept through the middle point of the line between the ischiatic tubercle and the greater trochanter of the femur, and presented oval or triangular high echo outside the biceps femoris and semitendinosus, that is, the sciatic nerve. The ultrasound guided puncture reached the sciatic nerve, and after the blood was drawn back, 0.5% ropivacaine was slowly injected for 15 m | Lumbar epidural anesthesia was applied: Puncture was performed in the patient's L2~3 intervertebral space, into the dural space, and then inserted into the lumbar puncture needle. After seeing the outflow of cerebrospinal fluid, 5% ropivacaine 12~15 mg was slowly injected, and then an epidural catheter was applied, and appropriate anesthetic was injected as required. | Nausea, Vomiting, Urinary retention | NA | NA |
| **Deng Bin** | **2020** | **Retrospective study** | Brachial plexus nerve trunks (composed of C5 to T1 nerve roots) | Above the hyoid muscle, within the intermuscular groove of the anterior and middle oblique muscles | Nerve block anesthesia was performed under ultrasound guidance, SonoSite M-Turbo color ultrasound instrument, probe frequency of 5 ~ 10 MHz, intersulci brachial plexus nerve puncture. Before the puncture, the surrounding tissues, brachial plexus and blood vessels were examined by ultrasound. Inject 20mL mixture of 0.375% ropivacaine. After the drug enters all nerve branches, the puncture needle is removed and anesthesia is completed | Anesthesia was performed under routine anatomic positioning, and the puncture point was above the thyrohyoid muscle and inside the intermuscular groove of the anterior middle scalene muscle. Puncture with a 7-gauge needle, insert the needle 1.5cm to the side of the foot, and fix the needle. After withdrawal without bleeding, 30mL of 0.375% ropivacaine mixture was injected to complete anesthesia. | Nausea and vomiting, urinary retention, chills | NA | NA |
| **Kwon Young Sil** | **2020** | **Retrospective study** | Brachial plexus nerve trunks (composed of C5 to T1 nerve roots) | Above the thyrohyoid muscle, within the intermuscular groove of the anterior middle scalene muscle | Ultrasound-guided nerve block anesthesia was performed, color ultrasound instrument was selected to assist anesthesia operation, and the probe frequency was set at 5-10MHz. The brachial plexus, blood vessels and surrounding tissues were explored first, and then the brachial plexus nerve was punctured in the intermuscular groove, and the needle was injected with ropivacaine mixture under the guidance of ultrasound (Ychang Renfu Pharmaceutical Co., LTD.; Sinopod H20103636), the concentration was 0.375%, the injection dose was 30ml. After the anesthetic has entered each nerve branch, the puncture needle is pulled out | Anesthesia under routine anatomical positioning was performed, puncture was performed above the thyrohyoid muscle and between the muscular grooves of the anterior middle scalene muscle. Puncture was performed with a 7-gauge needle on the side of the inclined foot, the depth was controlled at 1.5cm, and the needle was fixed. If no bleeding was found after withdrawal, ropivacaine mixture was injected (Yichang Renfu Pharmaceutical Co., LTD.; Sinopod H20103636), the concentration was 0.375%, the injection dose was 30ml. | Chills, nausea and vomiting, urinary retention | NA | NA |
| **Zhang Aiping** | **2020** | **Retrospective study** | About 1 point below the midpoint of the dorsal side of the line connecting the highest points of the bilateral anterior superior iliac spines The puncture points are set at about 5 cm and 4 cm to the horizontal side of the block side | Puncture was performed at the L3-4 lumbar intervertebral space and spinal anesthesia was inserted The needle is inserted into the subarachnoid space after the cerebrospinal fluid is drained | In the same position as the control group, lumbar plexus block anesthesia was performed under the guidance of ultrasound. The highest point of the anterior superior iliac spine on both sides is connected with the median point of the back about 1. The puncture point was made at about 5 cm and 4 cm on the horizontal side of the block side, and the electrode was installed on the calf, assisted positioning was carried out through the neurostimulator, the frequency was set at 2 Hz, the stimulation current was 1 mA, the probe was perpendicular to the patient's skin, the needle tip was attached to the probe into the needle, and the needle was guided by ultrasound to the lumbar muscle space near the lumbar plexus. A typical contraction of the quadriceps signals a successful puncture, and the current is set to 0. At 4 mA, if the quadriceps was still contracted and no blood was drawn back, 30 mL of ropivacaine was given intravenatically. Sciatic nerve block anesthesia is as follows: The frequency of the probe was adjusted to 4 ~ 8 MHz, and the transverse scan was performed at the midpoint of the connection between the ischiatic tubercle and the greater trochanter of the femur. The puncture point was selected to be about 3 cm below the midpoint of the connection between the greater trochanter of the femur and the posterior superior iliac spine. The needle was guided by ultrasound into the psoas space near the sciatic nerve. 4 mA, if the above reaction is still present and no blood is drawn, 20 mL ropivacaine is given intravenously | A healthy lateral position was taken, puncture was performed in the L3 ~ 4 lumbar intervertebral space, lumbar anesthesia needle was inserted, cerebrospinal fluid was extracted and then injected into subarachnoid space. 5% Ropivacaine (Chengdu Baiyu Pharmaceutical Co., LTD., SinopOD H20183392) 1. 6 ~ 2. 0 mL, remove the lumbar anesthesia needle, place the epidural catheter to the head side, and wait for over 1. At 5 h, 2% lidocaine (Jiangsu Langou Pharmaceutical Co., LTD., National drug approval number H32023259) was added to the anesthetic plane for maintenance. | Nausea and vomiting, bradycardia, urinary retention, chills | Age ≥60 years old; X-ray and CT examination confirmed the femoral intertrochanteric fracture. All were treated with artificial femoral head replacement. All patients and their families give informed consent and sign informed consent forms. | Accompanied by severe liver, kidney, heart and other organ dysfunction; Blurred consciousness; Multiple site fracture; Associated with hematological diseases; With autoimmune diseases; Combined with severe hypertension and diabetes; Surgical contraindications exist; Refused to cooperate with the study. |
| **Yang Xianzhou** | **2017** | **Retrospective study** | Location of lumbar plexus block: A sagittal scan at the L3 level of the midline shows the space between the quadratus lumborum and the psoas major muscle. A needle is inserted in the plane 4 cm beside the midline into the space between the psoas major muscle. Location of sciatic nerve block The sciatic nodule was swept across the line connecting the greater trochanter of the femur, showing the sciatic nerve (oval with high echo), and then a puncture was performed for drug injection. | Lumbar plexus block The L4 spinous process is inserted 5 cm laterally and vertically. The space between the psoas major muscles is located when the resistance disappears. Sciatic nerve block The needle is inserted vertically at the midpoint of the line connecting the ischial tuberosity and the greater trochanter of the femur, and the location is determined by abnormal sensation. | Ultrasound-guided lumbar plexus combined with sciatic nerve block anesthesia, the patient was placed in lateral position, and the ultrasound low-frequency Sonoacoustic S-Nerve probe was applied. The images of the quadrate and psoas major muscles of the patient were displayed satisfactorily at L3 ~ 5 level using paracentral and sagittal techniques and transverse scanning. Local infiltration anesthesia of 1% lidocaine was administered about 4 cm outside the midline, intraplane injection was applied, the injection was stopped when the tip reached the space of the psoas major muscle, and 0.05% ropivacaine was slowly injected with 20 ml. Ultrasound was used to sweep the line between the ischial tubercle and the greater trochanter of the femur. After satisfactory images were obtained, in-plane needle puncture was performed beside the sciatic nerve and 0.05% ropivacaine 25 ml was injected. | The patient was instructed to take a lateral position and given local infiltration anesthesia with 1% lidocaine. The needle was opened 5 cm beside the L4 spinal process, and the needle was inserted vertically through the quadrate of the lumbar muscle between the upper and lower transverse processes into the interpsoas sulci. After the resistance was removed, 0.05% ropivacaine 20 ml was slowly injected. The sciatic nerve was blocked and the central point of the line between the sciatic tubercle and the greater trochanter was vertically injected into the needle until the pain was relieved and then 0.05% ropivacaine 25 ml was injected. | not clear | NA | NA |
| **Chen Rong** | **2018** | **Retrospective study** | Location of lumbar plexus block: Horizontal scan at L4 level shows the L4 transverse process, erector spinae, quadratus lumborum and psoas major muscle structures. Epidural puncture is performed right beside the midline of the spine, with ultrasound guiding the needle tip to the nerve. Location of sciatic nerve block The midpoint of the line connecting the sciatic nodule and the greater trochanter of the femur was swept and located, and the puncture was performed beside the sciatic nerve. | Lumbar plexus block The L4 spinous process is inserted 4 to 5 cm vertically, and the psoas major muscle space is located when the resistance disappears. Sciatic nerve block The needle is inserted vertically between the ischial tuberosity and the greater trochanter of the femur, and the location is determined by abnormal sensation. | First, the domestic Minay Doppler ultrasound diagnostic instrument was used to transversally scan the L4 level, and 1% lidocaine was used for local anesthesia when the location was to [4 transverse process, vertical spinal muscle, quadrate muscle and psoas major muscle]. Epidural puncture was performed according to the guidance of ultrasound, which was located in the middle of the spine in the L4 space. The ultrasound showed that the human needle was stopped near the nerve at the tip of the needle, and the needle core was pulled out. When there was no blood return or cerebrospinal fluid discharge, 20 mL 0.5% ropivacaine was injected. Then, the ischial tubercle and the greater trochanter of the femur were scanned by ultrasound, and the intermediate point was located for puncture. The puncture depth was near the sciatic nerve, and 25 mL of 0.5% ropivacaine was injected. | The patient was kept on his side, L4 position was determined, and 1% lidocaine was injected for local anesthesia. After that, the anesthesia needle was inserted into the center of the spine on the L4 side at the position of 4 ~ 5era. After the needle was inserted into the transverse process, it was slightly inclined upward, and the needle depth was 0.5 ~ 1cm. When there was no resistance, the needle core was pulled out, and when there was no blood return or cerebrospinal fluid outflow, 20mL 0.5% ropivacaine was gently injected. The needle was inserted vertically 1 ~ 2 cm between the greater trochanter of the femur and the Angle of the sacrum, and 25 mL 0.5% ropivacaine was injected when different sensations appeared. | Lower limb paresthesia, urinary retention, bradycardia | NA | NA |
| **Yu Chunlei** | **2019** | **Retrospective study** | Lumbar plexus nerve block: Approach at the intermuscular space of the lumbar region; The scanning point is set at the intersection of the line connecting the midline of the spine and the line connecting the highest points of the iliac bones on both sides (if a hypoechoic area appears, puncture will be performed at this position). Sciatic nerve block: The approach below the gluteus maximus;The scanning point is the area of the connection between the ischial tuberosity and the greater trochanter of the femur (the sciatic nerve is triangular in ultrasound image with the tip upward). | Puncture point: 5cm lateral to the midline of the spine at the L4 space (determined based on the iliac crest). Needle insertion point: 2cm below the sacral Angle between the greater trochanter of the femur (for sciatic nerve block). | Lumbar plexus sciatic nerve block anesthesia was guided by ultrasound. The patient was placed in lateral position with the affected limb on the top and flexed 30° toward the head. The lumbar plexus block anesthesia was administered through the approach at the position of the lumbar greater muscular space. The patient's lumbar plexus was scanned with a high-frequency ultrasound probe in the hospital, and the scanning point was set as the intersection point between the median line of the spine and the highest line of the iliac bone on both sides. If the patient had a low echo area, the needle was inserted at the corresponding position, and 5 mL ropivacaine (0.5%) was injected after no blood was drawn back, and the operation was completed after peripheral drugs infiltrated the patient's nerve bundle. If the patient has no adverse reactions after local anesthesia, continuous infusion of 20 mL of the above anesthetic drugs is required. The sciatic nerve block anesthesia was performed by the lower gluteus maximus approach. The ultrasound scanning point was the connecting area between the sciatic tubercle and the femoral greater trochanter. The ultrasound imaging of the sciatic nerve was triangular, the tip was upward, and high echo of the connective tissue was visible. The long axis of the ultrasonic probe and the long axis of the piercing needle are kept at 90°. Anesthesia drugs were injected around the sciatic nerve of the patient, and the total amount of drugs and lumbar plexus block anesthesia culture. | Epidural anesthesia was performed, and the patient was placed in the upper lateral position. The L4 position was determined based on the iliac crest, and local infiltration anesthesia was performed with lidocaine (1%). Epidural puncture was performed at the location 5 cm in the middle of the spine beside the L4 space, and the direction of needle insertion was kept vertical. After the needle reaches L4 transversal position, tilt slightly upward and insert 1.0 cm at the upper edge of transversal process. If the resistance disappears completely, the needle core can be pulled out. If there is no spinal fluid or blood return during the withdrawal process, slowly inject 20 mL ropivacaine (0.5%). The injection point was set to be 2cm below the greater trochanter of the femur and the Angle of the sacrum, and the injection direction was vertical. If there was foreign body sensation, 25 mL ropivacaine (0.5%) could be injected. During the operation, the anesthesia level should be controlled below T10. If the patient has hypotension during anesthesia, the infusion speed should be increased, and 6 mg ephedrine should be injected combined with the improvement of symptoms; if the patient has bradycardia during anesthesia, 0.5 mg atropine should be injected | Hypotension, bradycardia | NA | NA |
| **Huang Minzhen** | **2018** | **Retrospective study** | Ultrasound scan of the L4 transverse process → Insert the needle right beside the midline of the spine to the L4 nerve root. Ultrasound location: the midpoint of the line connecting the sciatic nodule and the greater trochanter of the femur, and puncture to the nerve | 5cm lateral to the L4 spinous process, puncture vertically to 2cm below the Angle between the greater trochanter of the femur and the sacrum in the psoas major muscle space, and puncture vertically to the point of sensation | The lower back plexus sciatic nerve block was guided by ultrasound for anesthesia. The L4 position of the patient was scanned by ultrasonic diagnostic instrument to determine the specific positions of quadrate lumbosus, psoas major muscle and L4 transverse process, and lidocaine was used for anesthesia. Assisted by ultrasonic diagnostic instrument, the medical staff carried out epidural puncture, and selected the patient to open the median spinal position beside the L4 space to inject the needle. After the tip of the puncture needle had reached the L4 nerve root, the injection was stopped, the needle core was pulled out, and the concentration of 0.5%ropivacaine was slowly injected into the patient from the puncture site with a dose of 20mL. Subsequently, ultrasound was used to scan the patient's ischial tubercle and the greater trochanter of femur, find the midpoint of the connection between the two, and perform puncture. The specific depth of puncture was to reach the sciatic nerve, and then 0.05% ropivacaine was slowly injected from the puncture site with a dose of 25mL. After the operation is completed, the clinical signs of the patient should be closely observed, and if there is any abnormal situation, relevant measures should be taken in time to deal with it | Anesthesia was performed with a conventional lumbar plexus sciatic nerve block. The specific method was as follows: the medical staff first placed the patient in the lateral position and placed the lower extremity requiring surgery on the top to facilitate the smooth operation. In the process of adjusting the position of the patient, the medical staff can give the patient a certain psychological counseling, inform them that they do not need to be nervous, there will not be too much pain during the operation, and determine the specific location of L4 in time. Lidocaine with 1% concentration (Manufacturer: Shanghai Pujin Linzhou Pharmaceutical Co., LTD.; Approval number: H41022244) for anesthesia, vertical puncture next to the L4 space, when the needle tip has reached the L4 transversal process, withdraw the needle a little and start to tilt upward, and enter the needle from the upper part of the transversal process, the depth is about 1cm. Subsequently, the needle core is pulled out and slowly injected with ropivacaine at a concentration of 0.5% (manufacturer :AstraZeneca AB; Approval No. : Import Drug Registration No. H20140763), the dose was 20mL, and the puncture was performed 2cm below the femur at the junction with the sacrum, and then 0.05% ropivacaine was injected after the puncture, the dose was about 25mL. | Inadequate anesthesia, puncture-related nerve injury, mild pain | NA | NA |
| **Yang Jie** | **2019** | **Retrospective study** | Puncture the midpoint of the line connecting the sciatic nodule and the greater trochanter of the femur to the nerve | Make a vertical puncture 2cm below the femur at the junction with the sacrum to the point of abnormal sensation | The ultrasound-guided lumbar plexus sciatic nerve block anesthesia was performed. Before anesthesia, ultrasound diagnostic instrument was used to scan the L4 position of the patient to determine the location of quadrate lumbosus, psoas major muscle and L4 transverse process. Then, local infiltration anesthesia was performed with 1% lidocaine, epidural puncture was performed with the assistance of ultrasound diagnostic instrument, and the needle was inserted into the median position of the spine beside the L4 space, and stopped when the puncture needle reached the L4 nerve root. Remove the needle core and inject 0.4% ropivacaine 20m l into the puncture site. In addition, the position of the ischial tubercle and the greater trochanter of the femur was determined by ultrasound diagnosis instrument, and the midpoint of the connection between the two was located for puncture. After the puncture needle reached the sciatic nerve, 25ml of 0.4% ropivacaine was injected. After the completion of anesthesia, closely observe the patient's vital signs, and deal with any abnormalities in time | Conventional lumbar plexus sciatic nerve block anesthesia was used. The patient was placed in a lateral position and the L4 position was determined by the iliac ridge point. First, 1% lidocaine was used for local infiltration anesthesia, and the puncture was performed vertically next to the L4 space. When the puncture needle reached the L4 transversal process, it was slightly inclined to the backward needle and continued to inject the needle 1cm above the transversal process, and then the needle core was pulled out and 0.4% ropivacaine was slowly injected 20m l. In addition, the puncture was performed at the junction of the lower femur 2c m and sacrum, and 0.4% ropivacaine 25ml was injected. | Mild pain, inadequate anesthesia | NA | NA |
| **Dong Dalong** | **2019** | **Retrospective study** | The nerve bundle between the sacrum and ilium | L2-3 or L3-4 intervertebral space puncture | The patients underwent ultrasound-guided lumbosacral plexus block combined with superficial general anesthesia of laryngeal mask, and L3 ~ was obtained. The puncture point was 4-5 cm beside the intervertebral space. The S.VE portable ultrasound instrument (3-6mhz low-frequency probe produced by sonosite in the United States) was used to guide the puncture. The probe plane was perpendicular to the spine, and an elliptical high-echo mass in the fascia of the psoas major was visible, that is, MJ at the location of the lumbar plexus. Lumbar plexus block was completed by injection of 20 mL 0.5% ropivacaine. The probe was moved to 1/3 of the line between the greater trochanter of the femur and the posterior superior iliac spine to show the position of the sacral plexus "J" between the sacrum and the iliac bone. The same procedure was performed by injection of 15 mL 0.5% ropivacaine to complete the sacral plexus block. After completion, the patient received intravenous injection of Sufentanil (Yichang Renfu Pharmaceutical Co., LTD., 2 mL: 100 bucket g) 0.1 ¨g/kg, propofol (Guangdong Jiabol Pharmaceutical Co., LTD., H20051842, 20 mL: 200 mg)2 mg/kg, after the patient's bi frequency index (BIS) reached 65, a human laryngeal mask was placed, and propofol was injected intravenously during the operation to maintain anesthesia. All patients in the 2 groups were treated with intravenous controlled analgesia (PCA) after surgery, the analgesia pump was indwelled for 48 h, and the drugs used were Sufentanil 2 mg and haloperidol (Shandong Yijian Pharmaceutical Co., LTD., Sinopol H37020967, 2 mL: 5 mg)5 mg, diluted to 200 mL, a single PCA amount of 1 mL, locking time 10-15 min. | L: ~, or L3. Lumbar epidural anesthesia "J "was performed in the intervertebral space, and 15 mL of 0.5% ropivaine (Xi 'an Libang Pharmaceutical Co., LTD., National drug approval number H20060475, 10 mL: 90 mg) was injected after successful puncture. An epidural catheter was indwelled after the completion of anesthesia, the patient was changed to a supine position, the anesthesia plane was observed, and T was reached within 15 minutes. The following can be, did not reach the epidural pursuit of calidocaine (Tianjin Pharmaceutical Group Xinzheng Co., LTD., Sinopod H41024475, 2 mL: 4 mg) 4-5 mL. | Nausea and vomiting, headache, agitation, hypotension | ① The structure or function of the hip joint is impaired and the patient is willing to undergo hip replacement surgery; American Society of Anesthesiologists (AsA) Levels I to II Ho; ③ Patient's informed consent | ① The patient's health cannot tolerate anesthesia or surgery; ② Patients with severe suppurative osteoarthritis. |
| **Cheng Yan** | **2017** | **Retrospective study** | Sacral plexus | L1-L2 transverse process gap | Patients in group C underwent epidural anesthesia in lateral position, and the puncture point was L1-2, the catheter was placed at a depth of 3cm. After lying flat, 3ml of 1% lidocaine was injected without blood, and the adverse reaction of local anesthetics was observed and the anesthetic effect was measured. 0.75% ropivacaine 8ml was given 5 minutes later. After 15 minutes, the anesthesia plane was detected by acupuncture method. During the operation, patients' vital signs were closely monitored and the amount of blood loss was recorded. According to the changes of vital signs, fluids and vasoactive drugs were timely treated, and blood transfusion was possible if necessary | The patient was placed in lateral position with the affected limb on top. An ultrasonic convex array probe (2~5MHz) was used as the puncture point next to the L2-L3 space. After detecting the image of the "palleti-type" transverse process, the needle was inserted in the middle of the transverse process space and 15ml of 0.5% ropivacaine was injected 1.5 ~ 2.0cm below the transverse process surface. The long axis of the ultrasound probe was placed within 1/2 of the intersection of the greater trochanter of the femur and the posterior superior iliac spine. It was observed that the posterior iliac probe continued to shift towards the ischiatic hole, and the posterior sacral plexus injection of 15ml 0.5% ropivacaine was detected. | Hypotension, urinary retention, nausea and vomiting, nerve injury, local anesthetic systemic toxicity (LAST) | Age 66~78 years old, ASA grade I ~ III. | There are obvious cardiopulmonary dysfunction, peripheral nerve disease, accompanied  Patients with any other contraindications to nerve blocking. |
| **Wei Nan Fu** | **2017** | **Retrospective study** | Lateral to the line connecting the posterior superior iliac spine and the ischial tubercle of the nerve bundle | L2-L3 transverse process gap | Ultrasound-guided femoral nerve combined with sciatic nerve block anesthesia: The ischial nerve block was performed by using the sonoro S-NERVE portable ultrasonic machine. After disinfection, the posterior superior iliac spine and the ischial tuberosity were marked, and a line was made between the two points. The probe was placed horizontally on the tailbone and laterally on the lateral part of the line. After images were obtained, 25 ml of 0.5% ropivacaine was injected into the sciatic nerve with lumbar anesthesia needle. After that, femoral nerve block was performed: the midpoint of the ventral groove on the affected side of the patient was taken, the femoral artery pulsation point was marked and disinfected, the probe was placed at the marked point for short-axis section scanning, and 20ml of 0.5% ropivacaine was injected around the femoral nerve with lumbar anesthesia needle. | Combined lumbar and epidural anesthesia was performed: L2-3 was selected as the puncture point for anesthesia puncture. The anesthetic agent was 2.0ml 0.75% bubicainine combined with 1.0ml 10% glucose. During the surgical treatment, 1.5% lidocaine was added epidural according to the actual situation (8-1) 0ml). | Nausea and vomiting, low back pain, headache | NA | NA |
| **Kateryna Bielka** | **2021** | **Retrospective study** | L2-L3 transverse process gap | L3-4 intervertebral space puncture | ultrasound-guided Shamrock CPB with bupivacaine 0.125% 6–8ml / h was performed. Intraoperative anes_x0002_thesia was provided with a bupivacaine bolus of 0.5% 200mg in a lumbar catheter and a sciatic nerve block (neurostimulator identifcation) with 1.5% 450mg of lidocaine. Postoperative analgesia include prolonged CPB with bupivacaine 0.125% 6–8ml / h. Also paracetamol 3g/day and dexketoprofen 75mg/day was prescribed | intraoperative spinal anaesthesia were per_x0002_formed at the level of L3-L4 with hyperbaric bupivacaine  10–15mg. Patients in groups 1 and 2 receive intraoperative sedation with propofol 1% with a target level of sedation RASS from 0 to −2. | Hypotension, myocardial injury after noncardiac surgery (MINS), nausea and vomiting, delirium | signed informed consent, age over 18years | patient age less than 18years, patient refusal, preg_x0002_nancy and lactation, history of opiate addiction, severe comorbidities (traumatic brain injury; acute stroke; dementia; acute cerebrovascular accident; chronic heart failure (New York Heart Association Functional Classifcation, NYHA, class III-IV), respiratory failure, renal failure with decreased creatinine clearance less than 30ml/min /1.73m2, hepatic insufciency class C according to  Child-Pugh) |
| **Yan Tang** | **2023** | **prospective study** | The line connecting the anterior inferior iliac spine and the pubic branch is placed horizontally. | NA | Te regional block was performed with the patient in the supine position. A curvilinear low-frequency ultrasound probe (2-6 MHz,Sonosite) was initially placed in a transverse plane over the anterior inferior iliac spine and then aligned with the pubic ramus by rotating the probe counterclockwise approximately 45 degrees. Te iliopsoas eminence, the iliopsoas muscle and tendon, the femoral artery, and the pectineus muscle were observed. A 22G puncture nee_x0002_dle was inserted from lateral to medial in an in-plane approach to place the tip in the musculofascial plane between the psoas tendon anteriorly and the pubic ramus posteriorly. Following negative aspiration, 2 mL of normal saline was injected to identify the correct location of the tip, followed by an injection of 20 mL of 0.375% ropivacaine (Naropin, AstraZeneca, eg, Fig. 1) | intravenous 50 mg of furbiprofen. | Nausea and vomiting | ① Patients with an imaging diag_x0002_nosis of hip fracture; ② Age ≥60 years old; ③ Dynamic  NRS scores ≥4 points; ④ BMI: 18-30 kg/m2 | ① Patients with allergies to the drug used in  this experiment; ② Patients with local or systemic infec_x005ftion; ③ Patients with coagulopathy; ④ Patients with  severe cardiopulmonary insufciency; ⑤ Patients with a  history of ipsilateral hip surgery; ⑥ Patients with mental, language, communication, or hearing impairment;  ⑦ Patients who refused to participate in this trial; today  Patients with multiple systemic injuries. |
| **Liang Jin** | **2020** | **prospective study** | The junction of the transverse process and the rib | NA | After placing the patient in the lateral decubitus position, the anesthesiologist discerned the puncture site with a linear 5 to 10MHz ultrasound probe (LOGIQe, GE Healthcare, Waukesha,WI) as follows: after distinguishing the targeted transverse process from the junction of the ribs on the horizontal plane, the cranial end of the transverse process was marked on the skin as the puncture site on the sagittal plane. After standard skin disinfection, a 17G Tuohy needle (Henan Tuoren Medical Device Co., Ltd., Xinxiang, Henan, China) was inserted perpendicularly or slightly caudally into the paravertebral space at the T4 or T5 level (Fig. 1A). A 20G catheter (Henan Tuoren Medical Device Co., Ltd., Xinxiang, Henan, China) was inserted up to the needle tip through the Tuohy needle, and then the Tuohy needle was withdrawn. The catheter was fixed to the skin and connected to a syringe. When aspiration demonstrated the absence of air or blood, 15 to 20mL 0.375% ropivacaine and 10mg sufentanil were injected into unilateral blocks before skin incision. Ten milliliters of 0.375% ropivacaine and 10mg of sufentanil were injected through the catheter every 6 hours. The catheter was withdrawn 48 hours after the operation | . All patients were intravenously administered 0.4m g/kg sufentanil again when the thoracic incision was closed.Sufentanil and tropisetron were used for patient-controlled analgesia (PCA) within 48hours post-surgery (continuous infusion with sufentanil 0.05mg/kg/h and a bolus dose with sufentanil 0.03mg/kg and a lock time of 15 minutes). A numerical rating scale (NRS) score from 0 to 10 was used to assess pain in all patients at rest and coughing for two days after surgery. Surgeons performed all esophagectomies via posterolateral thoracotomy. A single chest drain was placed in the sixth intercostal space in the mid-axillary line. The chest drain was removed 48hours after surgery according to the volume of drainage and lung recruitment. | Postoperative atelectasis, nausea and vomiting, pruritus, delirium | e patients aged 65 to 75 years who underwent elective esophagectomy for stage III and IV esophageal cancer | allergic reactions to local anesthetic; brain injury or neurosurgery; cardiovascular or cerebrovascular disease; chronic obstructive pulmonary disease; neurological or psychiatric disorders; drug and alcohol abuse; hepatic and/or kidney dysfunction; BMI>35kg/m2; and inability to communicate. The included patients were randomized to receive ultrasound-guided continuous thoracic PVB before the induction of anesthesia or patient-controlled analgesia (PCA) at the end of the operation. The randomization was performed using an online randomization tool (http://www.randomization.com). |
| **Qiu Dongjie** | **2023** | **prospective study** | The T5 intervertebral space on the surgical side | NA | After induction, anesthesiologist performed unilateral, sin_x0002_gle injection TPVB at T5 level with ultrasonography (GE Healthcare®, Wisguidance, Wauwatosa, WI) in group T. A high-frequency linear ultrasonography (probe frequency 7.5–10 MHz, depth 4.5–5.5cm) was placed perpendicular to the dorsal midline on the target spinous process. The probe was moved to the affected side so that the screen showed both the target spinous process and the transverse process of the next stage. Move  the probe slightly to the cephalic side to avoid the next stage of the transverse process, that is, the probe is located between and parallel to the two transverse processes, and the gap between the deep and lateral facet (about 1cm) and the pleura is the paraspinal thoracic space. Peripheral block needle (Stimuplex® A; B Braun, Melsungen, Germany) was inserted from the outside of the probe, avoiding the pleura, using in-plane technique. The needle was placed in the paraspinal thoracic space during  injecting 20mL 0.375% ropivacaine to ensure that the pleura was moved down by the drug solution, indicating drug diffusion in the intervertebral space. Local anesthetic distribution above pleura was checked by moving the probe up and down to confirm.[9–11] After the operation, patients’ vital signs, tidal volume, airway pressure, hipoxemia events and other respiratory parameters were observed. | Upon arrival at the operating room, patients were monitored with 5-lead ECG, pulse oximetry (SpO2), invasive blood pres_x0002_sure (IBP), end-tidal carbon dioxide and BIS as standard ASA monitoring. Anesthesia induction was the same for all groups with propofol (1.5–2mg/kg), sufentanyl (0.5–1 µg/kg) and double lumen tube (DLT) insertion facilitated with cisatracurium (0.2mg/kg). The left position of DLT was confirmed with a fiberoptic bronchoscope. Anesthesia maintenance was achieved with sevoflurane in air-oxygen mixture and remifentanil (0.1–0.2 µg/kg/min), cisatracurium was administered intermittently to maintain muscle relaxation, to maintain the BIS index between 45 and 55. All patients received 1 to 2 µg/kg additional doses of fentanyl per hour and 0.1mg/kg loading dose of morphine prior to thoracic closure. Perioperative adverse events (hypotension, bradycardia) were recorded. | Hypotension, respiratory depression, atelectasis, nausea and vomiting, delirium | NA | chronic pain and opioid using, local anesthetic allergy,  spinal deformity, communication difficulties, psychiatric disor_x0002_der, and Mini-mental State Examination < 25. |
| **Jianhong Hao** | **2019** | **Retrospective study** | The potential gap between the iliac fascia and the iliopsoas muscle | NA | Continuous FICB was performed under ultrasound guidance by the same anesthesiologist. A total of 30 ml (0.45%)ropivacaine solution was infused. Then an electronic pump was connected to the catheter with 200 ml (0.9%) sodium chloride (NaCl) at a concentration of 0.25% ropivacaine and a speed of 6 ml/h. | using 0.9% sodium chloride. On the ward, when patients suffered from severe pain (visual analogue scale [VAS] over 5), fentanyl 0.05 mg intramascular was given each time by the surgeon. The surgeon was blinded to the study | delirium | NA | NA |

NOTE: NA means not clear
