# Supplementary material for: Efficacy and safety of ultrasound-guided nerve blocks in elderly surgical patients: a meta-analysis
Source: Front Med (Lausanne). 2025 Aug 18;12:1580172. doi: 10.3389/fmed.2025.1580172 (PMC12399598; doi:10.3389/fmed.2025.1580172)
Supplement: Supplementary file 2 [file Supplementary_file_2.docx]

Supplementary file 2


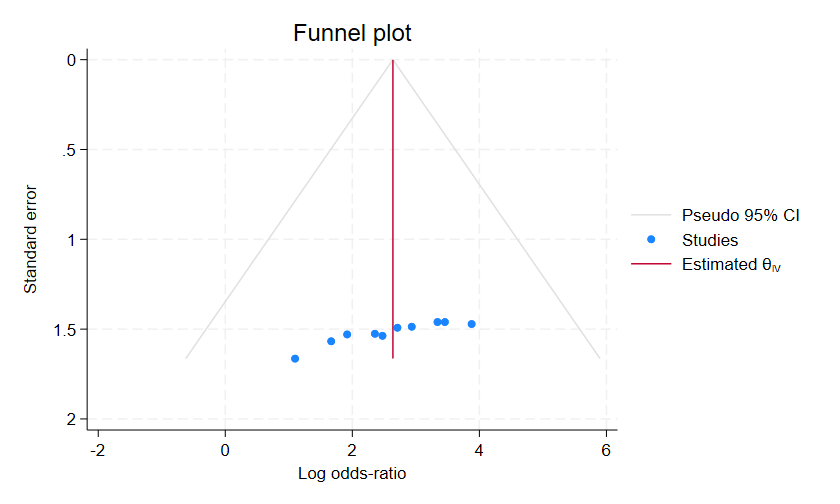


Figure 1 Funnel diagram of the Effective rate

| Table 1 The Effective rate of Sensitivity Analysis Results | | | |
| --- | --- | --- | --- |
| Study omitted | Estimate | 95% CI | |
| Zhang Weiqi (2019) | 1.21 | 1.09 | 1.34 |
| Wang Yuansheng (2016) | 1.24 | 1.12 | 1.38 |
| Li Fangqing (2017) | 1.22 | 1.10 | 1.36 |
| Yu Changwei (2018) | 1.21 | 1.09 | 1.34 |
| Deng Bin (2020) | 1.24 | 1.12 | 1.38 |
| Yang Xianzhou (2017) | 1.21 | 1.09 | 1.35 |
| Huang Minzhen (2018) | 1.20 | 1.09 | 1.33 |
| Yang Jie (2019) | 1.22 | 1.09 | 1.36 |
| Dong Dalong (2019) | 1.24 | 1.11 | 1.39 |
| Kateryna Bielka (2021) | 1.18 | 1.09 | 1.28 |
| Combined | 1.22 | 1.10 | 1.34 |


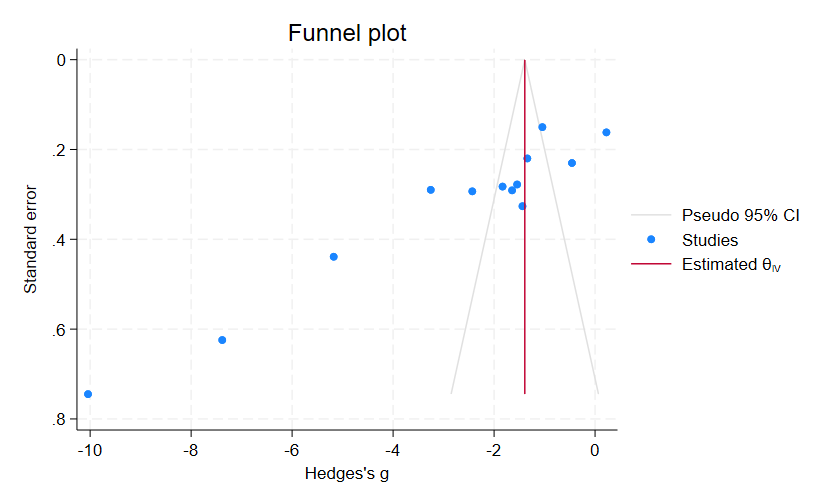


Figure 2 Funnel diagram of sensory nerve block onset time

| Table 2 The Sensory nerve block onset time of Sensitivity Analysis Results | | | |
| --- | --- | --- | --- |
| Study omitted | Estimate | 95% CI | |
| Bian Yupu(2020) | -2.21 | -3.02 | -1.41 |
| Wang Zepeng(2022) | -2.97 | -3.95 | -1.98 |
| Zhang Xi(2020) | -2.38 | -3.24 | -1.52 |
| Deng Bin(2020) | -2.85 | -3.83 | -1.86 |
| Kwon YoungSil(2020) | -2.87 | -3.86 | -1.88 |
| Zhang Aiping(2020) | -2.71 | -3.65 | -1.77 |
| Yang Xianzhou(2017) | -2.90 | -3.91 | -1.89 |
| Chen Rong(2018) | -2.88 | -3.85 | -1.90 |
| Yu Chunlei(2019) | -2.94 | -4.00 | -1.87 |
| Huang Minzhen(2018) | -2.54 | -3.43 | -1.65 |
| Yang Jie(2019) | -2.86 | -3.85 | -1.88 |
| Cheng Yan(2017) | -2.79 | -3.76 | -1.82 |
| Wei NanFu(2017) | -3.01 | -3.92 | -2.09 |
| Combined | 2.76 | 3.67 | 1.85 |


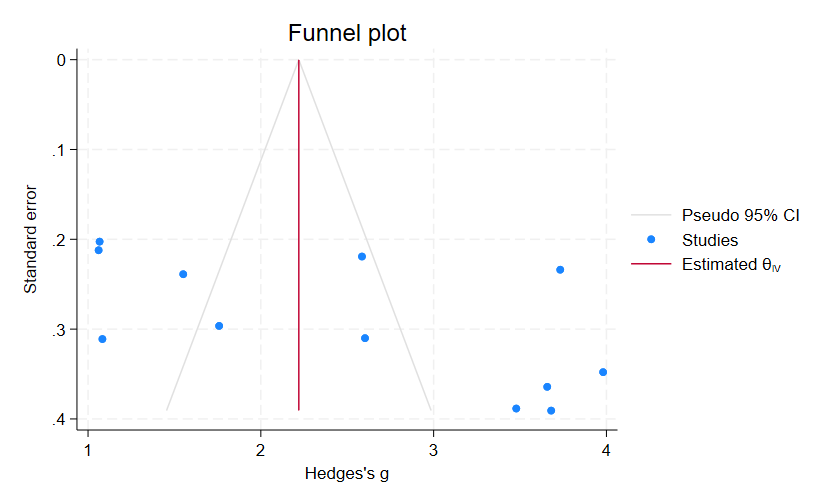


Figure 3 Funnel diagram of sensory nerve block duration time

| Table 3 Sensory nerve block duration time of Sensitivity Analysis Results | | | |
| --- | --- | --- | --- |
| Study omitted | Estimate | 95% CI | |
| Bian Yupu (2020) | 2.39 | 1.72 | 3.05 |
| Wang Zepeng (2022) | 2.51 | 1.80 | 3.23 |
| Deng Bin (2020) | 2.42 | 1.74 | 3.10 |
| Kwon Young Sil (2020) | 2.43 | 1.75 | 3.12 |
| Zhang Aiping (2020) | 2.66 | 1.98 | 3.33 |
| Yang Xianzhou (2017) | 2.66 | 1.98 | 3.34 |
| Chen Rong (2018) | 2.65 | 1.96 | 3.34 |
| Yu Chunlei (2019) | 2.40 | 1.76 | 3.05 |
| Huang Minzhen (2018) | 2.61 | 1.89 | 3.33 |
| Yang Jie (2019) | 2.59 | 1.87 | 3.31 |
| Cheng Yan (2017) | 2.42 | 1.74 | 3.10 |
| Wei Nan Fu (2017) | 2.52 | 1.78 | 3.26 |
| Combined | 2.52 | 1.86 | 3.18 |


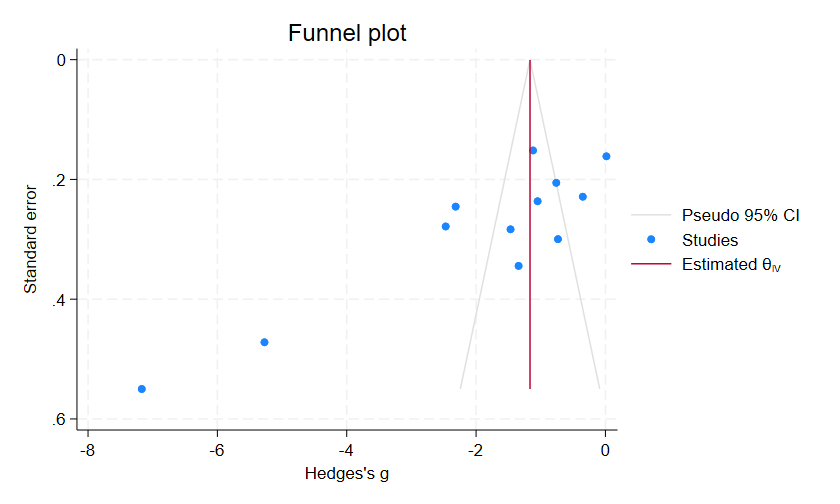


Figure 4 Funnel diagram of motor nerve block onset time

| Table 4 The Motor nerve block onset time of Sensitivity Analysis Results | | | |
| --- | --- | --- | --- |
| Study omitted | Estimate | 95% CI | |
| Bian Yupu (2020) | 1.49 | 2.11 | 0.88 |
| Wang Yuansheng (2016) | 1.99 | 2.79 | 1.19 |
| Wang Zepeng (2022) | 2.09 | 2.89 | 1.28 |
| Zhang Xi (2020) | 1.63 | 2.32 | 0.95 |
| Zhang Aiping (2020) | 1.90 | 2.69 | 1.12 |
| Yang Xianzhou (2017) | 2.06 | 2.88 | 1.23 |
| Chen Rong (2018) | 2.05 | 2.85 | 1.25 |
| Yu Chunlei (2019) | 2.03 | 2.90 | 1.16 |
| Huang Minzhen (2018) | 1.88 | 2.66 | 1.11 |
| Yang Jie (2019) | 1.98 | 2.79 | 1.18 |
| Cheng Yan (2017) | 2.03 | 2.84 | 1.21 |
| Wei Nan Fu (2017) | 2.12 | 2.89 | 1.35 |
| Combined | 1.94 | 2.68 | 1.19 |


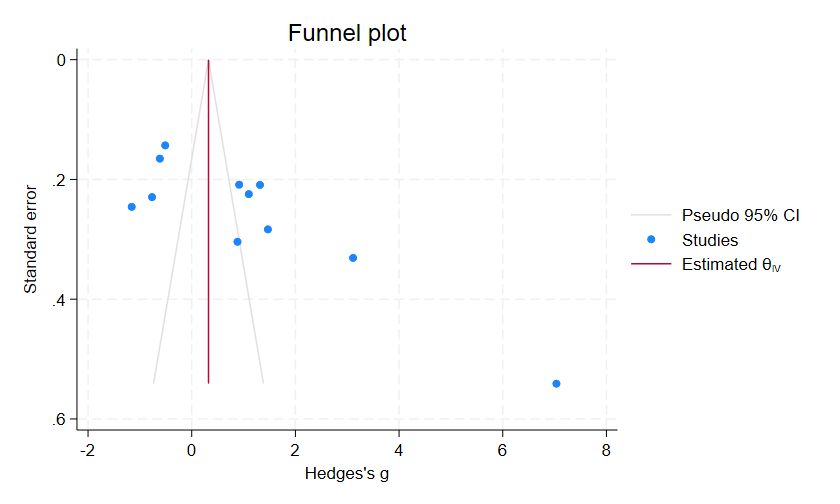


Figure 5 Funnel diagram of motor nerve block duration time

| Table 5 Motor nerve block duration time of Sensitivity Analysis Results | | | |
| --- | --- | --- | --- |
| Study omitted | Estimate | 95% CI | |
| Bian Yupu (2020) | 0.56 | 0.16 | 1.29 |
| Wang Zepeng (2022) | 1.34 | 0.43 | 2.25 |
| Zhang Xi (2020) | 0.90 | 0.05 | 1.75 |
| Zhang Aiping (2020) | 1.09 | 0.15 | 2.03 |
| Yang Xianzhou (2017) | 1.13 | 0.17 | 2.10 |
| Chen Rong (2018) | 1.13 | 0.20 | 2.07 |
| Yu Chunlei (2019) | 1.28 | 0.31 | 2.26 |
| Huang Minzhen (2018) | 1.11 | 0.16 | 2.06 |
| Yang Jie (2019) | 1.07 | 0.15 | 2.00 |
| Cheng Yan (2017) | 1.30 | 0.37 | 2.24 |
| Wei Nan Fu (2017) | 1.29 | 0.33 | 2.25 |
| Combined | 1.11 | 0.24 | 1.98 |


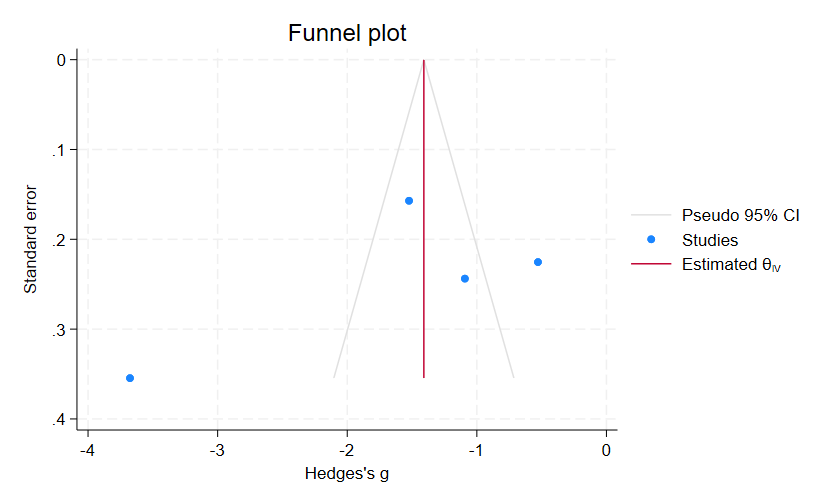


Figure 6 Funnel diagram of VAS score post operation 4H

| Table 6 VAS score postoperation 4H of Sensitivity Analysis Results | | | |
| --- | --- | --- | --- |
| Study omitted | Estimate | 95% CI | |
| Wang Zepeng (2022 | -1.89 | -3.29 | -0.49 |
| Deng Guohua (2022 | -2.08 | -3.28 | -0.87 |
| Qiu Dongjie (2023 | -1.76 | -3.39 | -0.13 |
| Jianhong Hao (2019 | -1.07 | -1.67 | -0.47 |
| Combined | -1.68 | -2.68 | -0.68 |


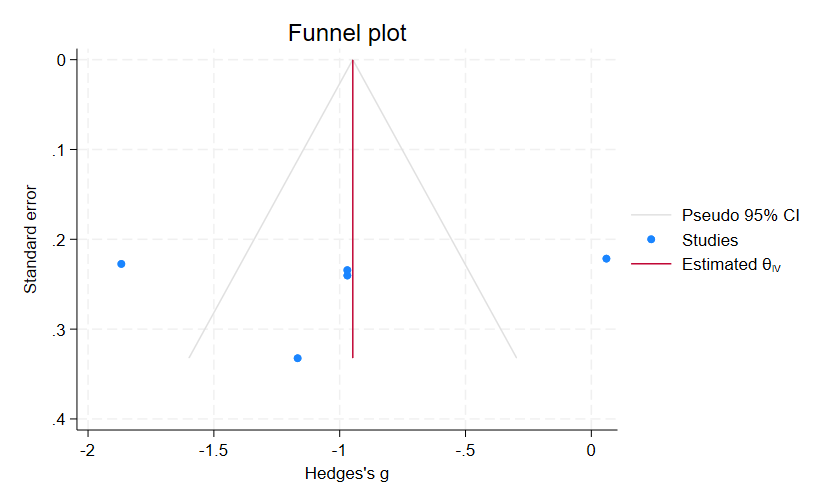


Figure 7 Funnel diagram of VAS score post operation 8H

| Table 7 VAS score post operation 8H of Sensitivity Analysis Results | | | |
| --- | --- | --- | --- |
| Study omitted | Estimate | 95% CI | |
| Wang Zepeng (2022) | -0.99 | -1.86 | -0.12 |
| Deng Guohua (2022) | -0.99 | -1.87 | -0.12 |
| Zhang Aiping (2020） | -0.75 | -1.34 | -0.17 |
| Dong Dalong (2019） | -1.26 | -1.73 | -0.80 |
| Yan Tang (2023） | -0.94 | -1.74 | -0.14 |
| Combined | -0.99 | -1.66 | -0.32 |


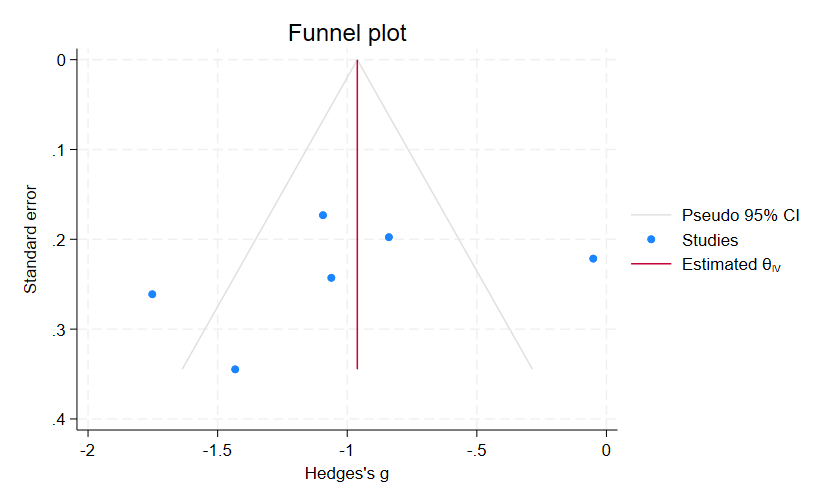


Figure 8 Funnel diagram of VAS score post operation 12H

| Table 8 VAS score post operation 12H of Sensitivity Analysis Results | | | |
| --- | --- | --- | --- |
| Study omitted | Estimate | 95% CI | |
| Wang Zepeng (2022） | -1.02 | -1.57 | -0.48 |
| Deng Guohua (2022） | -0.88 | -1.31 | -0.45 |
| Zhang Aiping (2020） | -1.07 | -1.64 | -0.51 |
| Dong Dalong (2019） | -1.20 | -1.51 | -0.90 |
| Wei Nan Fu (2017） | -1.02 | -1.60 | -0.44 |
| Yan Tang (2023） | -0.96 | -1.45 | -0.46 |


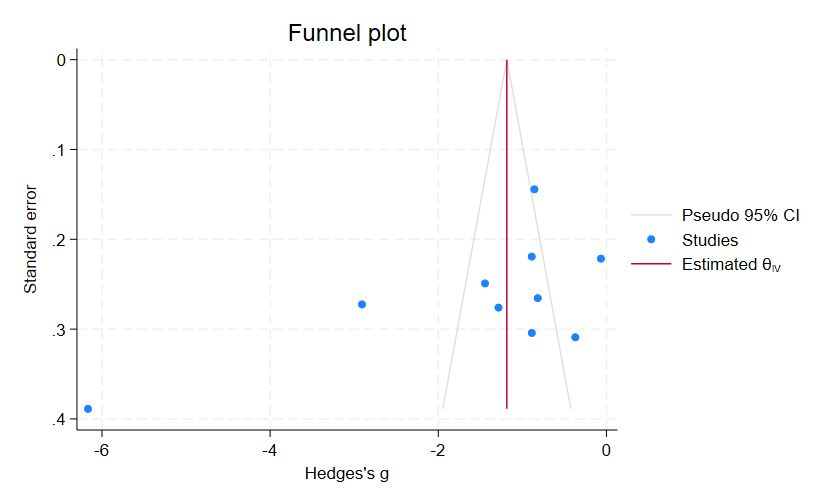


Figure 9 Funnel diagram of VAS score post operation 24H

| Table 9 VAS score postoperation 24H of Sensitivity Analysis Results | | | |
| --- | --- | --- | --- |
| Study omitted | Estimate | 95% CI | |
| Deng Guohua (2022) | 1.57 | -2.47 | -0.67 |
| Zhang Aiping (2020) | 1.40 | -2.21 | -0.59 |
| Chen Rong (2018) | 1.63 | -2.51 | -0.75 |
| Huang Minzhen (2018) | 1.64 | -2.55 | -0.72 |
| Yang Jie (2019) | 1.59 | -2.48 | -0.70 |
| Dong Dalong (2019) | 1.73 | -2.59 | -0.87 |
| Wei Nan Fu (2017) | 1.06 | -1.55 | -0.58 |
| Kateryna Bielka (2021) | -1.64 | -2.53 | -0.75 |
| Yan Tang (2023) | 1.69 | -2.56 | -0.82 |
| Qiu Dongjie (2023) | 1.64 | -2.61 | -0.67 |
| Combined | -1.56 | -2.36 | -0.75 |


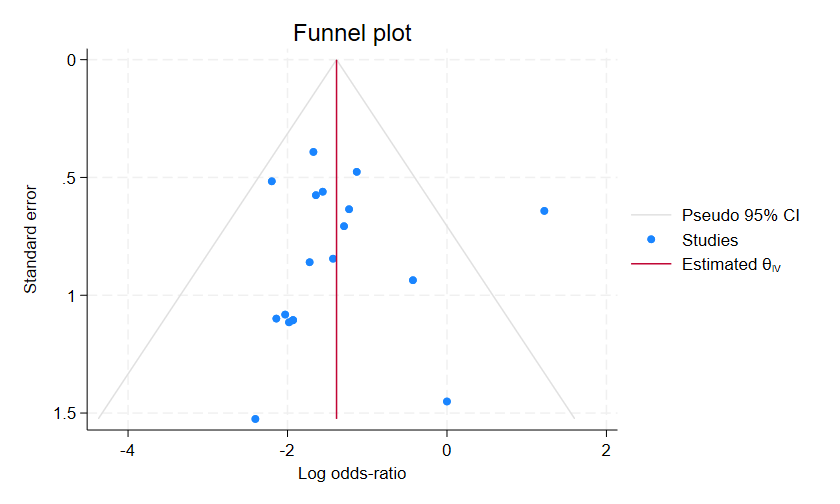


Figure 10 Funnel diagram of adverse reaction rate

| Table 10 Adverse reaction rate of Sensitivity Analysis Results | | | |
| --- | --- | --- | --- |
| Study omitted | Estimate | 95% CI | |
| Bian Yupu (2020) | 0.33 | 0.19 | 0.57 |
| Cao Yongchao (2021) | 0.35 | 0.27 | 0.44 |
| Wang Yuansheng (2016) | 0.33 | 0.19 | 0.57 |
| Wang Zepeng (2022) | 0.35 | 0.20 | 0.60 |
| Deng Guohua (2022) | 0.34 | 0.19 | 0.60 |
| Li Fangqing (2017) | 0.35 | 0.20 | 0.60 |
| Yu Changwei (2018) | 0.35 | 0.20 | 0.60 |
| Zhang Xi (2020) | 0.35 | 0.20 | 0.60 |
| Deng Bin (2020) | 0.35 | 0.20 | 0.61 |
| Kwon Young Sil (2020) | 0.34 | 0.20 | 0.59 |
| Zhang Aiping (2020) | 0.33 | 0.18 | 0.59 |
| Chen Rong (2018) | 0.34 | 0.20 | 0.60 |
| Dong Dalong (2019) | 0.34 | 0.19 | 0.59 |
| Cheng Yan (2017) | 0.34 | 0.19 | 0.60 |
| Wei Nan Fu (2017) | 0.35 | 0.20 | 0.60 |
| Kateryna Bielka (2021) | 0.32 | 0.17 | 0.59 |
| Liang Jin (2020) | 0.34 | 0.19 | 0.60 |
| Jianhong Hao (2019) | 0.34 | 0.19 | 0.59 |
| Combined | 0.34 | 0.20 | 0.58 |


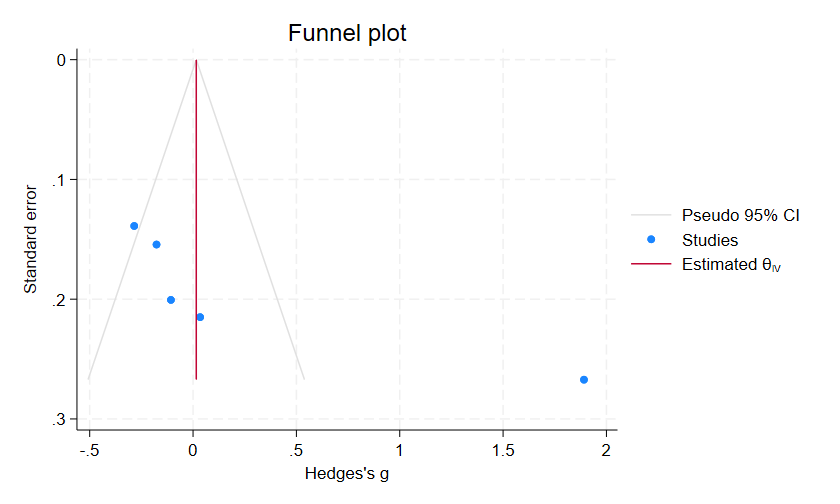


Figure 11 Funnel diagram of operating time

| Table 11 Operating time of Sensitivity Analysis Results | | | |
| --- | --- | --- | --- |
| Study omitted | Estimate | 95% CI | |
| Bian Yupu (2020) | 0.34 | -0.43 | 1.12 |
| Dong Dalong (2019) | -0.17 | -0.34 | -0.01 |
| Liang Jin (2020) | 0.37 | -0.46 | 1.19 |
| Qiu Dongjie (2023) | 0.39 | -0.41 | 1.20 |
| Jianhong Hao (2019) | 0.31 | -0.46 | 1.07 |
| Combined | 0.25 | -0.36 | 0.86 |
